# Web-Based Cognitive Behavior Therapy for Depression in People With Diabetes Mellitus: A Randomized Controlled Trial

**DOI:** 10.2196/jmir.7274

**Published:** 2017-05-15

**Authors:** Jill Newby, Lisa Robins, Kay Wilhelm, Jessica Smith, Therese Fletcher, Inika Gillis, Trevor Ma, Adam Finch, Lesley Campbell, Gavin Andrews

**Affiliations:** ^1^ School of Psychology The University of New South Wales Randwick Australia; ^2^ The University of New South Wales at St Vincent's Hospital Clinical Research Unit for Anxiety and Depression School of Psychiatry Darlinghurst Australia; ^3^ St Vincent's Health Australia Faces in the Street Darlinghurst Australia; ^4^ St Vincent's Hospital, Sydney Consultation Liaison Psychiatry Darlinghurst Australia; ^5^ Garvan Institute, St Vincent's Hospital, Sydney Diabetes Centre Darlinghurst Australia; ^6^ School of Psychiatry Clinical Research Unit for Anxiety and Depression The University of New South Wales at St Vincent's Hospital Darlinghurst Australia

**Keywords:** depression, diabetes mellitus, cognitive behavior therapy, diabetes-related distress, computer-assisted therapy

## Abstract

**Background:**

Depression is twice as common in diabetes mellitus (DM) as the general population and is associated with adverse health outcomes, but access to evidence-based therapies such as cognitive behavioral therapy (CBT) is limited in routine diabetes care. Past research has shown that generic Internet-based cognitive behavioral therapy (iCBT) is an effective treatment for depression in the general population, but it has never been evaluated in people with comorbid depression and DM.

**Objective:**

The aim of our study was to examine the efficacy of a generic 6-lesson iCBT delivered over 10 weeks in people with major depressive disorder (MDD) and DM.

**Methods:**

Participants with comorbid MDD and DM (type 1 or 2) were recruited online and randomized to an iCBT program with therapist support provided by phone and email (n=42) or a treatment as usual (TAU, n=49) control group. Outcomes were assessed through Web-based self-report questionnaires and the trial was Web-based with no face-to-face components. Primary outcomes were self-reported depression (patient health questionnaire-9, PHQ-9), diabetes-related distress (problem areas in diabetes, PAID), and self-reported glycemic control (hemoglobin A1c, HbA1c). Secondary outcomes were general distress (Kessler 10-item psychological distress scale, K-10) and disability (short form 12-item, SF-12), generalized anxiety (generalized anxiety disorder 7-item, GAD-7), and somatization (PHQ-15). The iCBT group was assessed at 3 months.

**Results:**

A total of 27 participants (66%; 27/41) completed the iCBT program. Analyses indicated between-group superiority of iCBT over TAU at posttreatment on PHQ-9 (*g*=0.78), PAID (*g*=0.80), K-10 (*g*=1.06), GAD-7 (*g*=0.72), and SF-12 mental well-being scores (*g*=0.66), but no significant differences in self-reported HbA1c levels (*g*=0.14), SF-12 physical well-being, or PHQ-15 scores (*g*=0.03-0.21). Gains were maintained at 3-month follow-up in the iCBT group, and the 87% (27/31) of iCBT participants who were interviewed no longer met criteria for MDD. Clinically significant change following iCBT on PHQ-9 scores was 51% (21/41) versus 18% (9/49) in TAU.

**Conclusions:**

iCBT for depression is an efficacious, accessible treatment option for people with diabetes. Future studies should explore whether tailoring of iCBT programs improves acceptability and adherence, and evaluate the long-term outcomes following iCBT.

**Trial Registration:**

Australian and New Zealand Clinical Trials Registry (ACTRN): 12613001198718; https://www.anzctr.org.au/Trial/Registration/TrialReview.aspx?id=365208&isReview=true (Archived by WebCite at http://www.webcitation.org/6qCR8Fi9V)

## Introduction

Diabetes mellitus (DM) is one of the largest causes of global health burden, affecting an estimated 415 million adults, with prevalence rates expected to rise to 643 million by 2040. The burden of this disease is significant in personal as well as economic terms, accounting for 5 million deaths, and 12% of global health care expenditure [1]. Depression is twice as common for people with DM compared with the general population [2], and places a substantial burden on people living with the disease, both in terms of personal suffering, and contributing to a range of adverse mental and physical health outcomes. Experiencing depression in the context of DM is associated with a range of adverse health outcomes, including poorer quality of life [3], higher diabetes-related distress [4], and poorer diabetes self-management, including lower adherence to self-care regimes (eg, physical activity and diet) [5]. Depression in DM is also associated with increased health care utilization and expenditure (eg, [6]), poorer medical outcomes, including worse glycemic control [7], and increased risk for diabetes-related complications and early mortality [8].

Effective management of depression is essential to reduce individual suffering and prevent these adverse outcomes [9]. Studies show that screening for depression has little influence on depression outcomes [10], and that proactive detection and treatment of depression is essential to reduce the burden of depression in people living with DM. A range of proactive treatment approaches have been found to be effective for treating depression in DM, including collaborative care [11], pharmacological interventions such as selective serotonin reuptake inhibitors (SSRIs), and psychological treatments [12]. Of the available psychological treatments, cognitive behavioral therapy (CBT) has the most empirical support for treatment for depression in DM. Meta-analyses show that CBT achieves clinically significant reductions in depression symptoms and improved quality of life [12]. Leading clinical practice guidelines therefore recommend routine assessment, screening, and treatment of depression in patients with DM [13], but despite these recommendations, depression is both underrecognized and undertreated: in routine care for DM, depression remains untreated in 50% of patients [6]. We now need new ways to deliver evidence-based depression interventions to people with DM, and overcome barriers underlying the shortfall of depression treatment in DM care, such as the lack of integration of mental and physical health services, and limited access to clinicians with expertise in addressing mental health issues in DM.

Delivering evidence-based psychological treatments via the Internet has potential to overcome some of these barriers. The efficacy of therapist-supported iCBT is now well established for the treatment of depression and anxiety disorders in the general population [14], and these positive effects generalize to routine care [15]. iCBT programs are now successfully being used to encourage proactive self-management of a range of chronic health conditions including chronic pain and irritable bowel syndrome [16]. iCBT has been shown to be as efficacious as face-to-face CBT [17], but can be delivered at a fraction of the cost and clinical time [18]. In contrast to pharmacotherapies that often have significant side effects [19], iCBT does not lead to harm, and deterioration in symptoms is rare [20].

Despite the potential for delivering mental health treatments on the Web to depression in people with DM, there are only two published randomized controlled trials (RCTs) examining the impact of Web-based programs for the treatment of depression in people with DM. In a sample of 225 Dutch adults with elevated depression (Center for Epidemiologic Studies Depression Scale [CES-D] scores >16) and type 1 (T1) or type 2 (T2) DM, van Bastelaar and colleagues found that a guided Web-based CBT program for depression with DM-specific content was more effective than treatment as usual (TAU) in reducing depression symptoms, increasing depression remission rates, and reducing diabetes-related distress with small-to-moderate between-groups effective sizes at 1-month follow-up (*d*=0.29 for intention-to-treat [ITT] analyses) [21]. The second trial with 260 German adults with T1 and T2 DM and elevated depression (CES-D ≥23), found that a guided Web-based intervention to reduce depression with DM-specific content was effective in reducing both depressive symptoms (ITT *d*=0.89) and diabetes-specific emotional distress (*d*=0.56) compared with a brief unguided Web-based psychoeducation program for depression [22].

These trials show promising results for using Web-based CBT programs to treat depression symptoms in people with DM, although it is not known whether results generalize to clinically depressed individuals with major depressive disorder (MDD). To our knowledge, there are no studies examining whether general or unmodified iCBT depression programs—without any content tailored to DM—are effective for people with DM. With generic evidence-based iCBT programs for depression now available for general public use, they present a novel opportunity to reduce the problem of depression in routine DM care, but need to be tested first to evaluate whether they are acceptable and effective in people with DM. Although RCTs have shown that face-to-face protocol-driven CBT interventions are effective for treating depression in DM, in-person CBT differs to iCBT in that it is able to be tailored and individualized to the individuals presenting problems. In contrast iCBT programs are typically highly standardized and fixed in format, and therefore need to be evaluated further in the context of DM.

This study is the first RCT to evaluate the efficacy of a generic clinician-guided iCBT program for MDD in people with T1 or T2 DM. The iCBT program has been previously demonstrated to be effective in RCTs [23] and effectiveness trials [15]. Whereas our primary aim was to examine the acceptability of the program for patients with DM and the efficacy of the iCBT program in reducing depression, we also sought to examine its impact on diabetes-related distress, generalized anxiety, mental and physical well-being, and glycemic control (hemoglobin A1c [HbA1c] levels). We hypothesized that adults with T1 or T2 DM who received iCBT would demonstrate significantly lower levels of depression, diabetes-related distress, and better self-reported glycemic control compared with the TAU control group at posttreatment.

## Methods

This study was approved by the St Vincent’s Hospital Human Research Ethics Committee (HREC/13/SVH/291). The trial was registered with the Australian and New Zealand Clinical Trials registry (ACTRN12613001198718).

### Design

Study details are reported in the published protocol [24]. Eligible participants were randomly allocated to the iCBT program or the TAU group who received the intervention after a 10-week waiting period. Simple randomization was used (1:1 ratio), and randomization numbers were generated by an independent research assistant using random.org. Group allocation was concealed in sequentially numbered opaque sealed envelopes. All participants provided electronic informed consent before participating.

### Eligibility

Inclusion criteria included: Australian resident, age 18 years or older, fluent in English, access to a computer and Internet, self-reported diagnosis of T1 or T2 DM, meet criteria for MDD according to telephone-administered diagnostic interview, and provide personal and general practitioner (GP) contact details. Participants were excluded if they had a self-reported diagnosis of bipolar affective disorder, psychotic disorder or substance use disorder, or were taking antipsychotics or benzodiazepines. Participants were excluded if they had commenced CBT in the past month, or changed antidepressant medication in the past 2 months. Participants scoring either <5 (normal range) or >23 (very severe) on the patient health questionnaire-9 (PHQ-9) were excluded, and those identified as being at significant risk of suicide or deliberate self-harm in the telephone risk assessment were also excluded and referred to appropriate treatments.

### Setting and Procedure

Participants were recruited from September 2013 to June 2015 by advertisements on DM websites, social media, and fliers. Interested applicants applied on the Web via the St Vincent’s Hospital, Sydney’s Virtual Clinic website, with an email and username, and completed Web-based screening questionnaires. Those who passed the Web-based screening criteria were assessed via telephone to confirm whether they met DSM-IV-TR diagnostic criteria [25] for MDD according to the Mini International Neuropsychiatric Interview (MINI) Version 5.0.0 [26]. There were no face-to-face components of the study.

### Interventions

#### Internet Cognitive Behavioral Therapy (CBT)

The iCBT Program is described in detail elsewhere [24,27] and a demo can be accessed by contacting the corresponding author. In brief, participants completed 6 automated cartoon-style Web-based lessons teaching CBT skills (eg, behavioral activation) over 10 weeks, with a minimum wait-time of 5 days between lessons. Participants downloaded a “homework” document which included practical assignments (eg, thought monitoring) after each lesson, and had access to extra resources, frequently asked questions, and recovery stories of former participants. Automated reminder emails were also sent to participants when lessons became available. Participants in the iCBT group were able to continue to receive usual care from their health services during the intervention period.

#### Treatment as Usual (TAU) Control Group

Participants in the TAU control condition waited 10 weeks to gain access to the iCBT program. During the waiting period, they were able to continue to receive usual care from their health services.

### Clinician-Guidance

Minimal clinician-assistance was provided to encourage adherence and engagement with the program by trained clinical psychologists with either Masters (LR) or PhD-level (JN) qualifications or psychiatry registrars (TM). Clinicians contacted the patient after lesson 1 and lesson 2 by email or phone to encourage progress. During the remainder of the program, clinician contact was made primarily by email, but if clinically indicated, or if patients’ Kessler 10-item psychological distress scale (K-10) or PHQ-9 scores deteriorated significantly, telephone contact was made by the clinician.

### Power Calculations

With a sample size of 40 participants per group, the study was powered (0.8 power) to detect a medium between-group difference of 0.65 on the primary depression measure at posttreatment (Cronbach alpha set at .05).

### Statistical Analyses

All analyses were undertaken in Statistical Package for the Social Sciences (SPSS) version 23 (IBM Corp, 2014). ITT linear mixed models analyses were used to account for missing data due to participant dropouts. This approach is appropriate for RCTs with multiple time points [28] and does not assume that the last measurement was stable (an assumption of the the last observation carried forward approach [29]). Linear mixed models were conducted separately for each of the dependent variable (DV) measures, with time, treatment group, and the time by group interaction entered as fixed factors in the model, with a random intercept for subject. For each outcome, an identity covariance structure was specified to model the covariance structure of the random intercept. Initial model building focused on the selection of the most appropriate covariance structure for the residual correlation matrix. Model fit indices and inspection of the variance-covariance matrix supported the selection of the identity covariance structure for each of the outcome measures. The fixed effect of age was added to each of the models. For each outcome measure except for problem areas in diabetes (PAID) scores, the fixed effect of age was not statistically significant and was removed from the model. Chi-square difference testing of the −2 log-likelihoods indicated that the removal of these fixed effects did not decrease model fit for any of the outcome variables, and they were excluded from further analyses.

For each group, planned contrasts were used to compare changes within and between groups from baseline to posttreatment (and 3-month follow-up for the iCBT group only). Between-group effect sizes using the pooled standard deviation and adjusted for sample size (Hedges *g*) were calculated to compare between groups at posttreatment. Within-group effect sizes (Cohen *d*) were calculated between pre- and posttreatment for both groups, and between pre- and 3-month follow-up for the iCBT group only. Effect sizes of 0.2, 0.5, and 0.8 were considered to be small, moderate, and large respectively in line with Cohen recommendations [30]. To investigate whether there were changes between posttreatment and follow-up for the iCBT group (n=21) for each dependent variable (eg, PHQ-9 scores), linear mixed models were conducted with time entered as a fixed factor and subject as a random intercept.

### Reliable Change

Reliable change index (RCI) values [31] were calculated for the PHQ-9 scores to determine the proportion of each group who evidenced reliable improvements (or deterioration) between baseline and posttreatment RCI values were calculated using test-retest reliability values of .84 from Kroenke et al (2001). In order to calculate standard error of measurement values, standard deviations were derived from current sample (PHQ-9 pretreatment pooled, SD 5.34). We compared the demographic and clinical characteristics of individuals in the iCBT group who were considered responders (reliable improvements in PHQ-9 scores) and nonresponders (no reliable change in PHQ-9 scores) using independent-samples *t* tests and chi-square for categorical data.

### Completers Versus Noncompleters

We also sought to compare the baseline demographic and clinical characteristics of individuals in the iCBT group who completed the entire program versus those who did not complete the program, using independent-samples *t* test and chi-square for categorical data.

### Measurements

All measures were Web-based self-report questionnaires, with the exception of the MINI diagnostic interview which was administered by telephone.

### Baseline Measures

At baseline, sociodemographic and sample characteristics were assessed including DM- and depression-related illness and treatment history.

### Primary Outcome Measures

The primary outcome measure was the PHQ-9 [32], which is a validated 9-item self-report measure of depression symptom severity over the past 2 weeks. The PHQ-9 contains items answered on a 4-point Likert scale; the total score ranges between 0 and 27 [33]. The PHQ-9 has been validated in diabetes samples [34].

Glycemic control was measured via self-reported HbA1c values. Diabetes-related distress was measured using the PAID [35] questionnaire, a well-validated 20-item measure with a 5-point Likert scale; total scores are multiplied by 1.25 and range from 0 to 100 (with higher scores indicating greater emotional distress). The PAID has demonstrated sensitivity to change [36] and good internal and test-retest reliability [35].

### Secondary Outcome Measures

The secondary outcome measures included the K-10 [37] for psychological distress; short form 12-item (SF-12) scale to measure of mental well-being (SF-12 MCS) and physical well-being (SF-12 PCS) [38], the generalized anxiety disorder 7-item (GAD-7) [39] for anxiety severity; and the PHQ-15—physical symptoms module for somatic symptom severity [40]. Other measures were also administered as part of the trial, which will be reported elsewhere. These include the relationships questionnaire (to assess attachment style), the alcohol and eating modules of the PHQ, and the fantastic checklist to assess specific lifestyle behaviours such as smoking status and alcohol use.

### Treatment Expectancy, Acceptability, and Satisfaction

Participants rated their expectancy of benefit from the intervention at baseline, and the acceptability and satisfaction with the program at posttreatment using the treatment credibility or expectancy questionnaire (CEQ) [41].

### Diagnostic Status

Current DSM-IV MDD diagnosis was assessed with the MINI version 5.0.0 [26] at baseline for both groups, and 3-month follow-up for the iCBT group only. The MINI possesses excellent interrater reliability (*k*=0.88-1.00) and good concurrent validity with the Composite International Diagnostic Interview (CIDI, World Health Organization, 1990) [42].

### Measurement Time-Points

Outcomes were assessed at 3 time points: baseline, posttreatment (11 weeks), and 3-month follow-up (for the iCBT group only, as the TAU group received the iCBT program after they completed the posttreatment assessment). The primary outcome measures (PHQ-9, PAID) were also administered at the mid-time point (5 weeks). Finally, the K-10 was administered prior to each lesson in the iCBT group to monitor distress.

## Results

### Participant Flow

**Figure 1:**
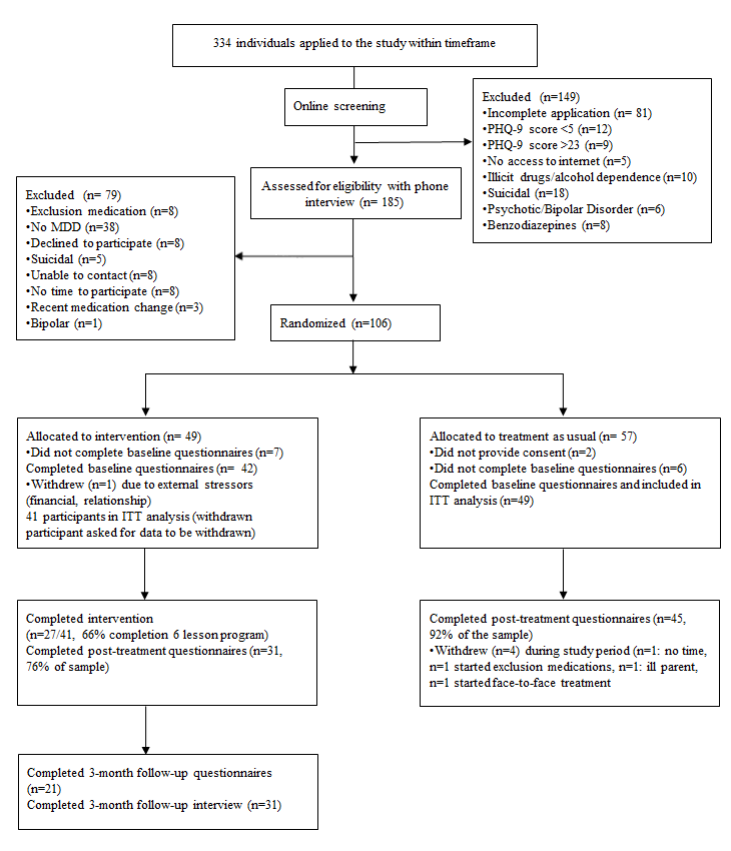
Consolidated standards of reporting trials (CONSORT) participant flow diagram. ITT: intention-to-treat; PHQ-9: patient health questionnaire-9.

Of the 334 individuals who started a Web-based application, 185 were eligible for phone interview. After phone interview, 106 individuals met the inclusion criteria and were randomized to either iCBT (n=49) or TAU (n=57). Of these participants, 42/49 allocated to the iCBT group and 49/57 allocated to the TAU group completed baseline assessment and were included in the ITT analysis. At posttreatment assessment, 31/49 provided data in the iCBT group and 46/49 provided data in the TAU group. At 3-month follow-up, 21 participants completed the questionnaires and 31 completed the diagnostic interview to assess MDD. See Figure 1 for study flow diagram.

### Baseline Characteristics

A total of 52 participants (57% of the sample) had T1 DM. Participants were 47 years on average (SD 12.61, range 20-71), and the majority were female (71%, 64/90) and married or living in a defacto relationship (55/90, 61%). Education status was mixed: one-fifth (16/90, 18%) had not completed high school, whereas 29 completed tertiary education (32%, 29/90). The majority were in full-time or part-time paid work (50/90, 56%), with 12 on the disability support pension (12/90, 6%). Only 16% (14/90) of the total sample were receiving psychological therapy, and 40% were taking medications for depression at baseline (37/90, 41%; see Table 1 for sample characteristics). Participants’ baseline depression levels were moderate to severe on the PHQ-9 (mean 15.0, SD 5.3). The majority reported 3 or more episodes of depression (80/90, 89%); two-thirds of the sample had not been depression free for at least 2 years (57/90, 63%), and half (46/90, 51%) reported being depressed for more than 4 years during their lifetime.

Almost three quarters of the sample (65/90, 72%) reported chronic disease comorbidities, with the average being 1.25 comorbid conditions (SD 1.07, range 0-4). Circulatory conditions were the most common (43/90, 48%), followed by arthritis conditions (30/90, 37%). It was found that 40% of the sample (n=37) reported having at least one DM-related complication, with eyesight problems the most common (n=14 individuals (16%, 14/90) followed by nerve damage (10/90, 11%).

### Baseline Between-Group Comparisons

There were no significant differences between the groups on baseline scores, the self-report measures (PHQ-9, PAID, K-10), and demographic variables (eg, DM type, gender, education, employment, depression onset, comorbid chronic conditions). The TAU group were older on average than the iCBT group (*t*_88_=2.22, *P*=.029).

### Adherence

It was found that 27 out of 41 individuals in the iCBT group completed all 6 lessons of the program, resulting in a 66% adherence rate. Of the noncompleters, 4 participants completed 1 lesson only, 3 completed 2 lessons, 2 completed 4 lessons, and 3 completed 5 lessons.

### Expectancy of Benefit

Prior to lesson 1, participants in the iCBT group were asked to provide a rating ranging from 1 to 9 about how logical the therapy offered to them seemed (where 1=not at all, 9=very logical), and how useful they thought the treatment would be in reducing their symptoms of depression (where 1=not at all, 9=very useful). The scores on these items were summed to derive an “expectancy of benefit” rating. On average, scores in the treatment group were positive (mean 11.27, SD 3.89, range 2-18).

### Primary Outcome Measures at Posttreatment and Effect Sizes

**Figure 2:**
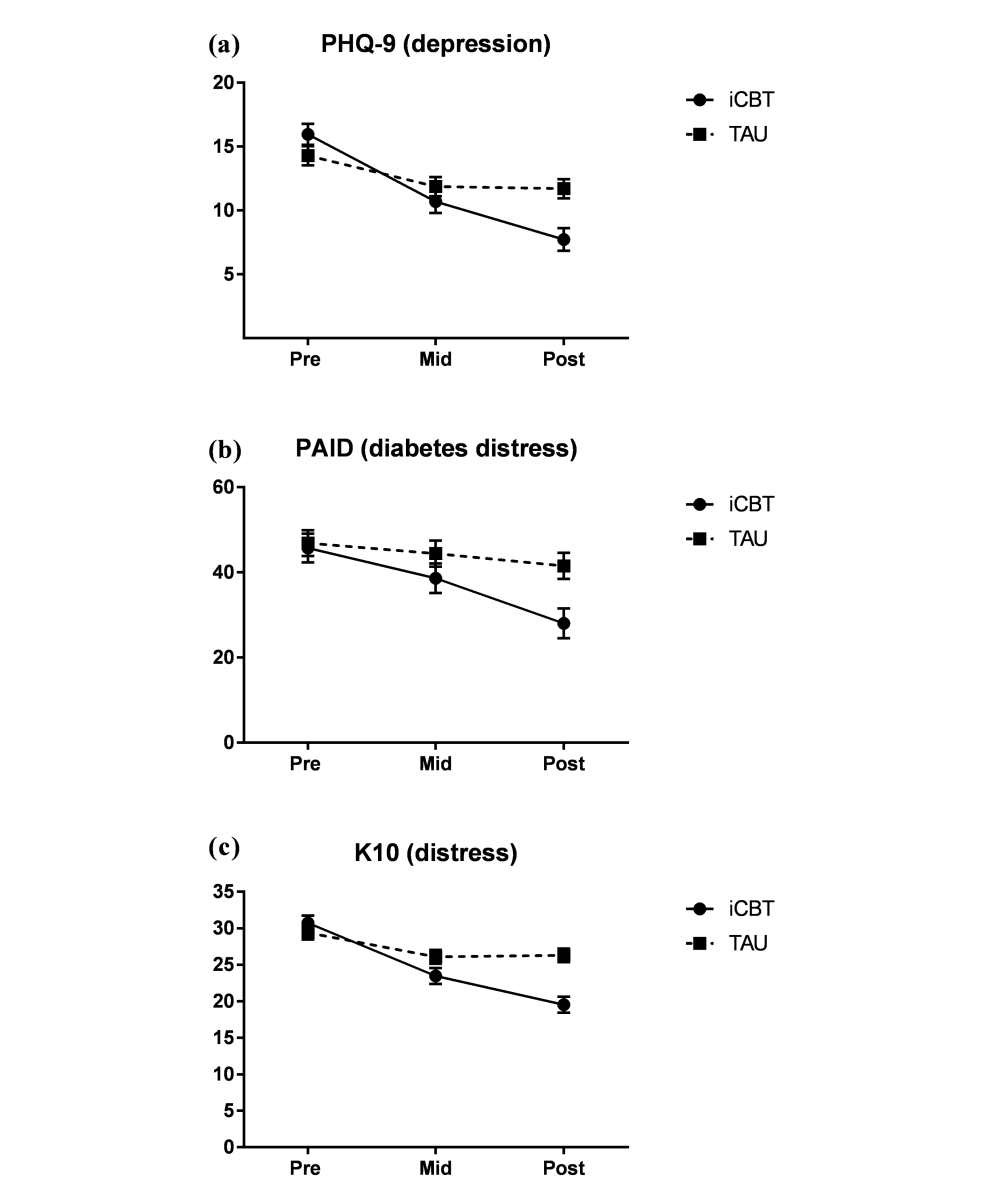
Estimated marginal means and standard errors for Internet-based cognitive behavioral therapy (iCBT) and treatment-as-usual (TAU) control group for (a) patient health questionnaire-9 (PHQ-9) (depression), (b) problem areas in diabetes (PAID) (diabetes-related distress), and (c) Kessler 10-item psychological distress scale (K-10) (general distress) at baseline, midtreatment, and posttreatment.

Table 2 includes estimated marginal means and linear mixed model results, and effect sizes for each of the outcome measures at baseline, midtreatment, and posttreatment. See Figure 2, PHQ-9 and PAID results. There were significant group by time interactions for the PHQ-9 (*F*_3,122.84_=10.41, *P*<.001), PAID (*F*_3,123.00_=10.32, *P*=.01), K-10 (*F*_3,153.20_=21.86, *P*<.001), SF-12 MCS scores (*F*_1,87.40_=9.07, *P*=.01), and GAD-7 scores (*F*_1,81.40_=13.18, *P*<.001). In contrast, the group by time interactions were not significant for SF-12 PCS (*F*_1,78.92_=0.27, *P*=.60), HbA1c levels (*F*_1,74.23_=0.11, *P*=.75), or PHQ-15 scores (*F*_1,86.59_=1.84, *P*=.18).

### Within-Group Effect Sizes (Baseline to Posttreatment)

Within-group comparisons for the iCBT group revealed large effect sizes between pretreatment and posttreatment on the PHQ-9 (*d*=1.90), PAID (*d*=1.18), K-10 (*d*=2.59), SF-12 mental well-being subscale (*d*=−0.85), and GAD-7 (*d*=1.38, 95% CI 0.86-1.90), and moderate for somatic symptom severity on the PHQ-15 (*d*=0.63). The changes on the SF12 physical well-being subscale and HbA1c levels were not significant (*P*>.05, see Table 2). The TAU group demonstrated medium statistically significant reductions in PHQ-9 (*d*=0.53), GAD-7 (*d*=0.42), and the K-10 (*d*=0.41), although the changes on the remaining outcome measures were not significant.

### Between-Group Effect Sizes (Posttreatment)

Posttreatment scores were significantly lower in the iCBT group relative to TAU on the PHQ-9, PAID, K-10, GAD-7, and SF-12 mental well-being subscales with moderate between-groups effect sizes on the PHQ-9 (*g*=0.78, 95% CI 0.30-1.25), GAD-7 scores (*g*=0.72, 95% CI 0.25-1.19), and SF-12 MCS scores (*g*=−0.66, 95% CI −1.12 to −0.19). In addition, we found large between-group differences for PAID scores (*g*=0.80, 95% CI 0.32-1.27) and K-10 (*g*=1.06, 95% CI 0.57-1.54). The between-group effect sizes for the other measures were small and not-significant (PHQ-15: *g*=0.21, 95% CI −0.24 to 0.67; HbA1c levels: *g*= *−* 0.14, 95% CI −0.62 to 0.34; SF-12 PCS: *g*=0.15, 95% CI −0.30 to 0.60).

**Table 1:** Baseline demographics and sample characteristics for the Internet-based cognitive behavioral therapy (iCBT) and treatment as usual (TAU) groups.

Variable		iCBT^a^group (n=41)	TAU^b^group (n=49)	Total (N=90)
**Diabetes type, n (%)**	
	Type 1	24 (59)	28 (57)	52 (58)
	Type 2	17 (42)	21 (43)	38 (42)
**Diabetes treatment, n (%)**			
	Insulin	29 (71)	34 (69)	63 (70)
	Diet alone	1 (2)	2 (4)	3 (3)
	Tablets	8 (20)	11 (22)	19 (21)
	Other	3 (7)	2 (4)	5 (6)
Age of onset (diabetes), mean (SD)		27.6 (18.1)	32.9 (16.2)	30.5 (17.2)
Diabetes complications (total), mean (SD)		0.83 (1.24)	1.15 (1.9)	1.0 (1.63)
Age (years), mean (SD)^c^		43.5 (13.3)	49.3 (11.5)	46.7 (12.6)
Baseline K-10^d^, mean (SD)		30.7 (5.9)	29.4 (6.7)	30.0 (6.4)
Baseline PHQ-9^e^, mean (SD)		15.95 (5.1)	14.3 (5.5)	15.0 (5.3)
Baseline PAID^f^, mean (SD)		38.1 (16.1)	36.5 (18.6)	37.2 (17.5)
**Gender, n (%)**	
	Male	8 (20)	18 (37)	26 (29)
	Female	33 (81)	31 (63)	64 (71)
**Marital status, n (%)**	
	Single or never married	9 (22)	10 (20)	19 (21)
	Married or defacto	29 (71)	26 (53)	55 (61)
	Separated or divorced or widowed	3 (7)	13 (27)	16 (18)
**Educational status, n (%)^i^**	
	Less than high school	6 (15)	10 (20)	16 (18)
	High school	3 (7)	5 (10)	8 (9)
	Tertiary (diploma)	6 (15)	8 (16)	14 (16)
	Tertiary (university degree)	11 (27)	10 (20)	21 (23)
	Tertiary (postgraduate degree)	5 (12)	3 (6)	8 (9)
	Other certificate	6 (15)	6 (12)	12 (13)
	Trade certificate	4 (10)	7 (14)	11 (12)
**Employment status, n (%)**	
	Full-time paid work	17 (42)	16 (33)	33 (37)
	Part-time paid work	5 (12)	12 (25)	17 (19)
	Unemployed	3 (7)	4 (8)	7 (13)
	Student	3 (7)	1 (2)	4 (4)
	Retired	5 (12)	0 (0)	12 (13)
	Disability support	5 (12)	7 (14)	12 (6)
	At home parent	3 (7)	2 (4)	5 (8)
**Age of onset (depression), n (%)**	
	Under 12	3 (7)	5 (10)	8 (9)
	13-21 years	14 (34)	15 (31)	29 (32)
	22 years or older	23 (56)	29 (59)	52 (58)
**Number of episodes (depression), n (%)**	
	1-2 episodes	5 (12)	5 (10)	10 (11)
	3-4 episodes	10 (24)	18 (37)	28 (31)
	5-8 episodes	10 (24)	6 (12)	16 (18)
	More than 8 episodes	16 (39)	20 (42)	36 (40)
**Total duration (depression) during lifetime, n (%)**	
	Less than 1 month	2 (5)	1 (2)	3 (3)
	1-12 months	10 (24)	9 (18)	19 (21)
	1-4 years	8 (20)	14 (29)	22 (24)
	More than 4 years	21 (51)	25 (51)	46 (51)
**Free of depression past 2 years, n (%)**	
	No	28 (68)	29 (59)	57 (63)
	Yes	13 (32)	20 (41)	33 (37)
**Comorbid chronic conditions, n (%)**	
	Asthma	13 (32)	15 (31)	28 (31)
	Cancer	2 (5)	4 (8)	6 (7)
	Stroke	2 (5)	1 (2)	3 (3)
	Circulatory condition	17 (42)	26 (53)	43 (48)
	Gout or rheumatism or arthritis	18 (44)	15 (31)	33 (37)
Current psychotherapy (psychology, social work, or counseling), n (%)		7 (17)	7 (15)	14 (16)
Current medication, n (%)		13 (32)	24 (50)	37 (41)
**Current medication (class), n (%)**	
	SSRI^g^	4 (10)	16 (33)	20 (22)
	SNRI^h^	8 (20)	8 (16)	16 (18)
	Other	1 (2)	0 (0)	1 (1)

^a^iCBT: Internet-based cognitive behavioral therapy.

^b^TAU: treatment as usual.

^c^Mean (SD): significant between-groups difference at *P*<.05 level.

^d^K-10: Kessler 10-item psychological distress scale.

^e^PHQ-9: patient health questionnaire-9.

^f^PAID: Problem areas in diabetes.

^g^SSRI: selective serotonin reuptake inhibitor.

^h^SNRI: selective noradrenaline reuptake inhibitor.

^i^Educational status: refers to the highest level of education received.

**Table 2:** Estimated marginal means (standard deviations) for primary and secondary outcome measures, within-group effect sizes, and between-group effect sizes.

Measure	Baseline Mean^a^ (SD)	Mid Mean^b^ (SD)	Post Mean^c^ (SD)	Within *t* (df) Baseline, Post	Within ES^d^ (95% CI) Baseline, Post	Between ES^e^ (95% CI) Post	*F* (time by group)
PHQ-9^f^ iCBT^g^	15.95 (5.25)	10.69 (4.98)	7.72 (4.96)	10.55 (159.32)	1.90 (1.34-2.45)	0.78 (0.30-1.25)	*F*_3,122.84_=10.41, *P*<.001
PHQ-9 TAU^h^	14.29 (5.25)	11.88 (5.15)	11.70 (5.15)	3.91 (152.40)	0.53 (0.12-0.94)	-	-
HbA1c^f^i iCBT	7.87 (1.79) or 63 mmol/mol	-	7.98 (1.73) or 64 mmol/mol	−0.48 (75.48)	−0.03 (−0.52 to 0.45)	−0.14 (−0.62 to 0.34), ns^j^	*F*_1,74.23_=0.11 *P*=.75, ns
HbA1c TAU	7.72 (1.82) or 61 mmol/mol	-	7.73 (1.76) or 61 mmol/mol	−0.05 (72.47)	-0.01 (−0.43 to 0.41)	-	-
PAID^k^ iCBT	45.73 (21.45)	38.65 (19.74)	28.00 (19.65)	7.74 (155.89)	1.18 (0.68-1.69)	0.80 (0.32-1.27)	*F*_3,87.03_=10.32, *P=*.01
PAID TAU	46.87 (21.28)	44.43 (20.82)	41.55 (20.82)	2.83 (152.09)	0.24 (−0.16 to 0.65), ns	-	-
K-10^l^ iCBT	30.73 (6.47)	23.46 (6.17)	19.54 (6.12)	12.03 (157.66)	2.59 (1.96-3.22)	1.06 (0.57-1.54)	*F*_2,155.20_=21.86, *P*<.001
K-10 TAU	29.39 (6.51)	26.10 (6.38)	26.28 (6.44)	3.99 (151.50)	0.41 (0.00-0.81)	-	-
SF-12 MCS^m^ iCBT	30.22 (10.12)	-	39.26 (9.80)	−5.38 (84.04)	−0.85 (−1.34 to −0.34)	−0.66 (−1.12 to −0.19)	*F*_1,87.40_=9.07, *P=*.01
SF-12 MCS TAU	29.79 (10.01)	-	32.70 (9.97)	−2.09 (77.55)	−0.27 (−0.68 to 0.13), ns	-	-
SF-12 PCS^n^ iCBT	40.15 (12.49)	-	41.1 (11.64)	−0.59 (80.95)	−0.08 (−0.55 to 0.38), ns	0.15 (−0.30 to 0.60), ns	*F*_1,78.92_=0.27, *P*=.60, ns
SF-12 PCS TAU	42.94 (11.27)	-	42.8 (11.12)	0.11 (75.97)	−0.01 (−0.39 to 0.41), ns	-	
GAD-7^o^ iCBT	10.73 (4.80)	-	4.60 (4.62)	7.21 (84.11)	1.38 (0.86-1.90)	0.72 (0.25-1.19)	*F*_1,81.40_=13.18, *P*<.001
GAD-7 TAU	10.11 (4.76)	-	8.02 (4.75)	2.97 (77.74)	0.42 (0.02-0.83)	-	
PHQ-15^p^ iCBT	11.90 (4.61)	-	8.65 (4.57)	3.23 (90.24)	0.63 (0.15-1.11)	0.21 (−0.24 to 0.67), ns	*F*_1,86.59_=1.84, *P*=.18, ns
PHQ-15 TAU	11.10 (4.55)	-	9.65 (4.68)	1.68 (81.88)	0.33 (−0.08 to 0.73), ns		

^a^Baseline: iCBT: n=41, TAU: n=49.

^b^Mid-treatment: iCBT: n=32, TAU: n=46.

^c^Posttreatment: iCBT: n=31, TAU: n=46.

^d^Within-group ES=Cohen *d.*

^e^Between-group ES=Hedges *g* with Hedges pooled SD.

^f^PHQ-9: patient health questionnaire-9.

^g^iCBT: Internet-based cognitive behavioral therapy group.

^h^TAU: treatment as usual group.

^i^HbA1c: haemoglobin A1c.

^j^ns: not significant, *P*>.05.

^k^PAID: problem areas in diabetes scale.

^l^K-10: Kessler 10-item psychological distress scale.

^m^SF-12 MCS: short form 12-item mental health subscale.

^n^SF-12 PCS: short form 12-item physical health subscale.

^o^GAD-7: generalized anxiety disorder 7-item scale.

^p^PHQ-15: patient health questionnaire 15-item somatization scale.

### Reliable Change

Of the iCBT group, 21/31 (51%) reliably improved compared with 9 (18%) in the TAU group. Of the iCBT group, no participants evidenced reliable deterioration, compared with only 2 participants in the TAU group (4%). The difference in proportions of participants who evidenced reliable change was significant (*χ*^2^_2_=18.4, *P<*.001). There were no significant differences between iCBT participants who showed reliable change versus those who did not show reliable change on any of the clinical or demographic variables at baseline (*t*=0.46-1.79, *P*>.08).

### Completers Versus Noncompleters in the Internet-Based Cognitive Behavioral Therapy (iCBT) Group

We compared baseline demographic and clinical characteristics of participants who completed all 6 lessons versus those who completed fewer than 6 lessons to explore whether there were any key differences at baseline in completers versus noncompleters. There were no significant differences on any of the clinical or demographic variables, including expectancy ratings at baseline. However, there was a trend toward higher K-10 distress scores in completers compared with noncompleters (K-10: completer sample (n=21): mean 31.96, SD 5.61, noncompleter sample (n=14): mean 28.36, SD 5.98, *t*_39_=1.91, *P*=.06, *g*=0.61, 95% CI 0.08-1.30).

### Outcomes for iCBT Group Between Posttreatment and 3-Months Follow-Up

There were no statistically significant effects of time for any of the outcome measures between posttreatment and follow-up (n=19). The within-group effect sizes were small and not significant (see Table 3).

### Diagnostic Status at Follow-Up

Of the total 31 participants in the iCBT group who completed a diagnostic interview to assess for MDD, 27 (87.1%) no longer met criteria for MDD.

### Clinician Time

The clinician spent on average 27.3 min per participant on email and telephone contact in the iCBT group (SD 14.9, range 8-71 min) over the course of the program. The clinician spent on average 13.51 min (SD 13.74, range 1-69 min) on the control group. This difference was significant (*t*_88_=4.55, *P*<.001).

### Patient Satisfaction

The iCBT participants were asked to provide a rating about: (1) how satisfied they were that the program taught them the skills to manage depression and (2) their confidence in recommending the program to a friend with similar problems (where 1=not at all, 5=somewhat, and 9=very). The overall mean scores were acceptable (satisfaction: mean 6.06, SD 2.14; recommend to friend (mean 6.84, SD 2.20, range 1-9). The majority of participants reported feeling somewhat to very satisfied with the program (n=27; 85%), although only 3 of these (9% of the sample collected at posttreatment) were “very” satisfied. The majority of participants reported feeling somewhat to very confident in recommending the program to a friend (n=28; 88%); of these 9 (28%) reported feeling very confident in recommending this program to a friend.

## Discussion

### Principal Findings

Our RCT in adults with T1 or T2 DM and MDD aimed to test whether a generic iCBT program for depression was more effective than usual care in improving depression. This is the first RCT to show that a generic iCBT program for the treatment of depression was superior to TAU in reducing depression, diabetes-related distress, anxiety, general distress, and improving mental well-being for people with comorbid T1DM or T2DM and MDD. On the primary outcome measure at posttreatment (PHQ-9), between-group effect sizes were moderate (*d*=0.78), and we also found large between-group differences at posttreatment on measures of diabetes-related distress (*g*=0.80) and general distress on the K-10 (*g*=1.06), and moderate between-groups effect sizes for generalized anxiety (*g*=0.72) and mental well-being (*g*=−0.66). These findings support the use of iCBT in the treatment of depression in DM.

Patients in the iCBT group experienced, on average, large reductions in depressive symptoms between baseline and posttreatment (ES=1.90). Benchmarked against previous studies of iCBT for depressive and anxiety disorders in the general population, these effect sizes are larger than those seen in patients who do not have chronic physical diseases [43]. At posttreatment, 51% of the iCBT group showed evidence of statistically reliable change compared with only 18% in the TAU group. There was minimal evidence of relapse between posttreatment and follow-up in the iCBT group, with 87% of the sample interviewed no longer meeting diagnostic criteria for MDD at 3-months follow-up. These findings are consistent with previous trials showing that iCBT leads to sustained improvements in depression symptoms beyond the completion of treatment, and even at long-term follow-up [44].

**Table 3:** Estimated marginal means (standard deviations) on primary and secondary outcomes between posttreatment and 3-month follow-up for the Internet-delivered cognitive behavioral therapy group.

Measure	Post mean (SD) T3^j^ (n=30)	3-month follow-up mean (SD) T4^k^ (n=19)	Within-group *t* (df) T3, T4	*r*	Within effect size^l^ (95% CI) T3, T4
PHQ-9^a^	10.09 (3.56)	10.98 (4.49)	−0.91 (27.53)	.68	−0.21 (−0.78 to 0.37), ns^b^
HbA1c^c^	7.84 (1.06)	7.71 (1.18)	0.48 (22.06)	.72	0.04 (−0.54 to 0.62), ns
PAID^d^	35.88 (14.19)	33.10 (16.17)	0.87 (22.18)	.76	0.17 (−0.41 to 0.74), ns
K-10^e^	23.69 (4.55)	23.20 (5.23)	0.47 (24.55)	.79	0.13 (−0.45 to 0.70), ns
GAD-7^f^	6.75 (2.85)	7.88 (3.57)	−1.45 (27.89)	.67	−0.34 (−0.93 to 0.22), ns
SF-12 MCS^g^	35.37 (7.07)	37.23 (10.11)	−0.77 (43.39)	.46	−0.16 (−0.73 to 0.42), ns
SF-12 PCS^h^	41.12 (7.01)	41.92 (8.24)	−0.47 (26.26)	.81	−0.09 (−0.66 to 0.49), ns
PHQ-15^i^	9.24 (2.79)	10.10 (3.66)	(22.49)	.86	−0.26 (−0.84-0.32), ns

^a^PHQ-9: patient health questionnaire-9.

^b^ns: not significant (*P*>.05).

^c^HbA1c: haemoglobin A1c.

^d^PAID: problem areas in diabetes.

^e^K-10: Kessler 10-item psychological distress scale.

^f^GAD-7: generalized anxiety disorder 7-item scale.

^g^SF-12 MCS: short form 12-item mental health subscale.

^h^SF-12 PCS: short form 12-item physical health subscale.

^i^PHQ-15: patient health questionnaire 15-item somatisation scale.

^j^T3: posttreatment.

^k^T4: 3-month follow-up.

^l^within-group ES=Hedges *g*.

Despite finding significant improvements in mental health and well-being, the positive effects of this program did not appear to translate to improved physical health outcomes or well-being. We failed to find a difference at posttreatment on the physical well-being subscale of the SF-12, somatic symptom severity on the PHQ-15, and self-reported HbA1c levels, and there was no evidence of improvement in self-reported physical health outcomes between posttreatment and 3-months follow-up in the iCBT group. Although these results may be due to lack of power, these preliminary findings suggest that although iCBT for depression improved mental health outcomes, at least in the short-term, it did not improve physical health outcomes. The relatively short follow-up period of this study is likely to have precluded us from finding a positive effect on physical health outcomes and self-management behaviors, which may only be observable at long-term follow-up [45].

This sample had poor health status, with 40% reporting one or more DM-related complications and 75% of the sample reporting one or more comorbid chronic diseases alongside DM. Despite living with severe and complicated physical health problems, participants in the iCBT group still reported better mental health at the end of treatment. Going forward, these improvements in mental health may help them to better cope with the challenges of managing diabetes and adjusting to changes in health by improving their resilience, or facilitating the use of positive coping strategies and self-care behaviors [46]. Further research is now needed to investigate whether improved physical health outcomes are observed at longer-term follow-up without any further intervention. We also recommend that future research focus on the mechanisms that underlie the positive changes we have observed in people with T1 or T2 DM and comorbid depression to better inform the development and tailoring of future treatments.

Overall, these findings are consistent with other studies showing that CBT for depression leads to improved depression outcomes, but has a limited effect on HbA1c levels [47]. The future challenge in this area of research is to identify treatment components and interventions that continue to positively impact on depression symptoms, but in addition derive beneficial changes in physical health outcomes, self-management, and biomarkers such as HbA1c levels. Diabetes distress is a broad-ranging construct covering many domains associated with living with diabetes, from adjustment to a new diagnosis through to the ongoing burden and burnout of managing a chronic condition. Although we noted large and significant reductions in diabetes distress as a consequence of our depression intervention, perhaps if diabetes distress were to be specifically targeted as part of the treatment, we would see broader improvements and even more significant change on this variable, with greater potential to impact on self-management behavior and physical outcomes. A multidisciplinary, multicomponent, Web-based treatment which addresses depression symptoms as well as the way that the emotional effects of living with diabetes can impact on self-care and self-management might be the key to seeing improvements in both mental and physical health.

We found only 66% of the participants completed the program and there was significant loss of data at follow-up (25% did not complete posttreatment assessments). Whereas these adherence rates are consistent with previous studies of Web-based depression management programs that incorporated diabetes-specific content in DM populations (eg, 62% adherence in 22), they are lower than rates of up to 80-90% found in previous trials of iCBT for depression and anxiety using the same protocols for providing clinician guidance to participants [48]. We failed to find any consistent differences between completers and noncompleters of the program in demographic characteristics, or baseline depression, anxiety, and diabetes distress severity, which may have been due to lack of power to detect key differences between these groups. Preliminary results suggested a trend toward higher distress scores in those who completed the program, although these findings need to be replicated in a larger sample.

Future research needs to determine the most effective ways to engage people with comorbid DM and depression in iCBT programs and examine the possible reasons for these lower adherence rates. First, it is possible that the program was not as acceptable for the participants who dropped out of the program, or may not have adequately addressed their unique concerns or difficulties that impacted on their depression symptoms. Although the general CBT skills may be useful and effective for improving depression for some individuals with DM, tailoring content to DM-related may improve the acceptability of the program for those who dropped out, and therefore improve engagement and completion rates. Second, in this study, participants had minimal guidance and monitoring from a therapist, but it is possible that more frequent or intensive guidance was needed to better engage the individuals who dropped out of the iCBT program. Therapist guidance during iCBT has been shown to promote adherence and influence program completion rates [49], although it is unclear how much, and what type of support is needed to achieve optimal adherence to iCBT interventions. Future research could benefit from examining differing levels of support on adherence. Finally, it is possible that the relatively low adherence rates in this study were reflective of broader issues with adherence in depressed DM populations [50]. Treatment adherence is an important consideration in diabetes self-management, with adherence to lifestyle, medication, and self-monitoring interventions critical to prevent the development of long-term complications, yet depressed individuals with DM have been shown to have poorer adherence to treatments in general [5].

### Limitations

The findings should be interpreted in the context of its limitations. Due to the fact that we do not have follow-up data in the TAU group, more research is needed to confirm the efficacy of iCBT for depression in people with DM in the long-term (eg, 6-12 months following treatment). Follow-up diagnostic interviews were not blinded to treatment allocation, introducing bias. The use of self-reported HbA1c levels to assess glycemic control was also a limitation of the study and we did not assess when the participants had their HbA1c levels tested. We chose this measure to mirror what occurs in usual clinical care because we wanted to increase the coverage of recruitment across Australia, and it was impractical to collect blood samples in other Australian states and territories. Finally, the high proportion of females and people with T1 DM may influence the generalizability of the findings.

### Conclusions

An unmodified Internet-delivered CBT program for depression is an effective intervention to improve depression and comorbid anxiety, general distress, and diabetes-specific distress in individuals with DM. Further research comparing unmodified versus tailored approaches for addressing depression in DM will clarify whether tailored approaches are more acceptable, engaging and therefore lead to better adherence rates. Given the increasing availability of evidence-based generic iCBT programs for depression in the general community and their ability to be implemented in routine care, they should be considered as a treatment option for those suffering from comorbid depression and DM. iCBT programs represent a scalable, accessible, evidence-based treatment option for people looking for effective treatment for their depression, who either are unable or do not wish to seek face-to-face psychological treatment.

## Abbreviations

CBT: cognitive behavioral therapy
CES-D: Center for Epidemiologic Studies Depression Scale
CEQ: credibility or expectancy questionnaire
CIDI: Composite International Diagnostic Interview
DM: diabetes mellitus
DV: dependent variable
GAD-7: generalized anxiety disorder 7-item
GP: general practitioner
iCBT: Internet-based cognitive behavioral therapy
K-10: Kessler 10-item psychological distress scale
MDD: major depressive disorder
MINI: Mini International Neuropsychiatric Interview
PAID: problem areas in diabetes
PHQ-9: patient health questionnaire-9
RCI: reliable change index
RCT: randomized controlled trial
SF-12: short form 12-item
SPSS: Statistical Package for the Social Sciences
SSRI: selective serotonin reuptake inhibitor
TAU: treatment as usual

## Appendix

Multimedia Appendix 1 CONSORT E-Health Checklist.
