# Supplementary material for: Web-Based Cognitive Behavior Therapy for Depression in People With Diabetes Mellitus: A Randomized Controlled Trial
Source: J Med Internet Res. 2017 May 15;19(5):e157. doi: 10.2196/jmir.7274 (PMC5447827; doi:10.2196/jmir.7274)
Supplement: Multimedia Appendix 1 [file jmir_v19i5e157_app1.html]

CONSORT-EHEALTH (V 1.6.1) - Submission/Publication Form


You are editing your previous response.

Be careful when sharing the URL of this page, because it will allow others to also edit your response.

Fill out a new response.

# CONSORT-EHEALTH (V 1.6.1) - Submission/Publication Form

The CONSORT-EHEALTH checklist is intended for authors of randomized trials evaluating web-based and Internet-based applications/interventions, including mobile interventions, electronic games (incl multiplayer games), social media, certain telehealth applications, and other interactive and/or networked electronic applications. Some of the items (e.g. all subitems under item 5 - description of the intervention) may also be applicable for other study designs.

The goal of the CONSORT EHEALTH checklist and guideline is to be   
a) a guide for reporting for authors of RCTs,   
b) to form a basis for appraisal of an ehealth trial (in terms of validity)

CONSORT-EHEALTH items/subitems are MANDATORY reporting items for studies published in the Journal of Medical Internet Research and other journals / scientific societies endorsing the checklist.

Items numbered 1., 2., 3., 4a., 4b etc are original CONSORT or CONSORT-NPT (non-pharmacologic treatment) items.   
Items with Roman numerals (i., ii, iii, iv etc.) are CONSORT-EHEALTH extensions/clarifications.

As the CONSORT-EHEALTH checklist is still considered in a formative stage, we would ask that you also RATE ON A SCALE OF 1-5 how important/useful you feel each item is FOR THE PURPOSE OF THE CHECKLIST and reporting guideline (optional).

Mandatory reporting items are marked with a red \*.   
In the textboxes, either copy & paste the relevant sections from your manuscript into this form - please include any quotes from your manuscript in QUOTATION MARKS,   
or answer directly by providing additional information not in the manuscript, or elaborating on why the item was not relevant for this study.

YOUR ANSWERS WILL BE PUBLISHED AS A SUPPLEMENTARY FILE TO YOUR PUBLICATION IN JMIR AND ARE CONSIDERED PART OF YOUR PUBLICATION (IF ACCEPTED).   
Please fill in these questions diligently. Information will not be copyedited, so please use proper spelling and grammar, use correct capitalization, and avoid abbreviations.

DO NOT FORGET TO SAVE AS PDF \_AND\_ CLICK THE SUBMIT BUTTON SO YOUR ANSWERS ARE IN OUR DATABASE !!!

Citation Suggestion (if you append the pdf as Appendix we suggest to cite this paper in the caption):  
Eysenbach G, CONSORT-EHEALTH Group  
CONSORT-EHEALTH: Improving and Standardizing Evaluation Reports of Web-based and Mobile Health Interventions  
J Med Internet Res 2011;13(4):e126  
URL: http://www.jmir.org/2011/4/e126/  
doi: 10.2196/jmir.1923  
PMID: 22209829

\* Required

Your name
\*

First Last


This is a required question

Primary Affiliation (short), City, Country
\*

University of Toronto, Toronto, Canada


This is a required question

Your e-mail address
\*

abc@gmail.com


This is a required question

Title of your manuscript
\*

Provide the (draft) title of your manuscript.

Web-based Cognitive Behaviour Therapy for depression in people with diabetes mellitus: a Randomised Controlled Trial.

This is a required question

Article Preparation Status/Stage
\*

At which stage in your article preparation are you currently (at the time you fill in this form)

- not submitted yet - in early draft status
- not submitted yet - in late draft status, just before submission
- submitted to a journal but not reviewed yet
- submitted to a journal and after receiving initial reviewer comments
- submitted to a journal and accepted, but not published yet
- published
- Other:

This is a required question

Journal
\*

If you already know where you will submit this paper (or if it is already submitted), please provide the journal name (if it is not JMIR, provide the journal name under "other")

- not submitted yet / unclear where I will submit this
- Journal of Medical Internet Research (JMIR)
- Other:

This is a required question

Manuscript tracking number
\*

If this is a JMIR submission, please provide the manuscript tracking number under "other" (The ms tracking number can be found in the submission acknowledgement email, or when you login as author in JMIR. If the paper is already published in JMIR, then the ms tracking number is the four-digit number at the end of the DOI, to be found at the bottom of each published article in JMIR)

- no ms number (yet) / not (yet) submitted to / published in JMIR
- Other:

This is a required question

## TITLE AND ABSTRACT

## 1a) TITLE: Identification as a randomized trial in the title

1a) Does your paper address CONSORT item 1a?
\*

I.e does the title contain the phrase "Randomized Controlled Trial"? (if not, explain the reason under "other")

- yes
- Other:

This is a required question

1a-i) Identify the mode of delivery in the title

Identify the mode of delivery. Preferably use “web-based” and/or “mobile” and/or “electronic game” in the title. Avoid ambiguous terms like “online”, “virtual”, “interactive”. Use “Internet-based” only if Intervention includes non-web-based Internet components (e.g. email), use “computer-based” or “electronic” only if offline products are used. Use “virtual” only in the context of “virtual reality” (3-D worlds). Use “online” only in the context of “online support groups”. Complement or substitute product names with broader terms for the class of products (such as “mobile” or “smart phone” instead of “iphone”), especially if the application runs on different platforms.

|  |  |  |  |  |  |  |
| --- | --- | --- | --- | --- | --- | --- |
|  | 1 | 2 | 3 | 4 | 5 |  |
| subitem not at all important |  |  |  |  |  | essential |

This is a required question

Does your paper address subitem 1a-i?
\*

Copy and paste relevant sections from manuscript title (include quotes in quotation marks "like this" to indicate direct quotes from your manuscript), or elaborate on this item by providing additional information not in the ms, or briefly explain why the item is not applicable/relevant for your study

Web-based Cognitive Behaviour Therapy for depression in people with diabetes mellitus: a Randomised Controlled Trial.

This is a required question

1a-ii) Non-web-based components or important co-interventions in title

Mention non-web-based components or important co-interventions in title, if any (e.g., “with telephone support”).

|  |  |  |  |  |  |  |
| --- | --- | --- | --- | --- | --- | --- |
|  | 1 | 2 | 3 | 4 | 5 |  |
| subitem not at all important |  |  |  |  |  | essential |

This is a required question

Does your paper address subitem 1a-ii?

Copy and paste relevant sections from manuscript title (include quotes in quotation marks "like this" to indicate direct quotes from your manuscript), or elaborate on this item by providing additional information not in the ms, or briefly explain why the item is not applicable/relevant for your study

The title is: Web-based Cognitive Behaviour Therapy for depression in people with diabetes mellitus: a Randomised Controlled Trial.
Although we have indicated the program is web-based, the actual telephone and email based support that was provided to participants was minimal (approximately 30 minutes per person throughout 12 weeks), so we have not included the additional support in the title as it was not the major focus of the intervention. 

This is a required question

1a-iii) Primary condition or target group in the title

Mention primary condition or target group in the title, if any (e.g., “for children with Type I Diabetes”) Example: A Web-based and Mobile Intervention with Telephone Support for Children with Type I Diabetes: Randomized Controlled Trial

|  |  |  |  |  |  |  |
| --- | --- | --- | --- | --- | --- | --- |
|  | 1 | 2 | 3 | 4 | 5 |  |
| subitem not at all important |  |  |  |  |  | essential |

This is a required question

Does your paper address subitem 1a-iii?
\*

Copy and paste relevant sections from manuscript title (include quotes in quotation marks "like this" to indicate direct quotes from your manuscript), or elaborate on this item by providing additional information not in the ms, or briefly explain why the item is not applicable/relevant for your study

Yes, see title.

This is a required question

## 1b) ABSTRACT: Structured summary of trial design, methods, results, and conclusions

NPT extension: Description of experimental treatment, comparator, care providers, centers, and blinding status.

1b-i) Key features/functionalities/components of the intervention and comparator in the METHODS section of the ABSTRACT

Mention key features/functionalities/components of the intervention and comparator in the abstract. If possible, also mention theories and principles used for designing the site. Keep in mind the needs of systematic reviewers and indexers by including important synonyms. (Note: Only report in the abstract what the main paper is reporting. If this information is missing from the main body of text, consider adding it)

|  |  |  |  |  |  |  |
| --- | --- | --- | --- | --- | --- | --- |
|  | 1 | 2 | 3 | 4 | 5 |  |
| subitem not at all important |  |  |  |  |  | essential |

This is a required question

Does your paper address subitem 1b-i?
\*

Copy and paste relevant sections from the manuscript abstract (include quotes in quotation marks "like this" to indicate direct quotes from your manuscript), or elaborate on this item by providing additional information not in the ms, or briefly explain why the item is not applicable/relevant for your study

Yes, see below from abstract.
Objective: To examine the efficacy of a generic 6-lesson internet-based Cognitive Behavioural Therapy for depression (iCBT) delivered over 10 weeks in people with major depressive disorder (MDD) and Diabetes Mellitus (DM).
Methods: Participants with comorbid MDD and DM (Type 1 or 2) were randomised to an iCBT program with therapist support provided by phone and/or email (n=42) or a treatment as usual control group (TAU, n=49). 

This is a required question

1b-ii) Level of human involvement in the METHODS section of the ABSTRACT

Clarify the level of human involvement in the abstract, e.g., use phrases like “fully automated” vs. “therapist/nurse/care provider/physician-assisted” (mention number and expertise of providers involved, if any). (Note: Only report in the abstract what the main paper is reporting. If this information is missing from the main body of text, consider adding it)

|  |  |  |  |  |  |  |
| --- | --- | --- | --- | --- | --- | --- |
|  | 1 | 2 | 3 | 4 | 5 |  |
| subitem not at all important |  |  |  |  |  | essential |

This is a required question

Does your paper address subitem 1b-ii?

Copy and paste relevant sections from the manuscript abstract (include quotes in quotation marks "like this" to indicate direct quotes from your manuscript), or elaborate on this item by providing additional information not in the ms, or briefly explain why the item is not applicable/relevant for your study

Yes, see below.
Objective: To examine the efficacy of a generic 6-lesson internet-based Cognitive Behavioural Therapy for depression (iCBT) delivered over 10 weeks in people with major depressive disorder (MDD) and Diabetes Mellitus (DM).
Methods: Participants with comorbid MDD and DM (Type 1 or 2) were randomised to an iCBT program with therapist support provided by phone and/or email (n=42) or a treatment as usual control group (TAU, n=49). 

This is a required question

1b-iii) Open vs. closed, web-based (self-assessment) vs. face-to-face assessments in the METHODS section of the ABSTRACT

Mention how participants were recruited (online vs. offline), e.g., from an open access website or from a clinic or a closed online user group (closed usergroup trial), and clarify if this was a purely web-based trial, or there were face-to-face components (as part of the intervention or for assessment). Clearly say if outcomes were self-assessed through questionnaires (as common in web-based trials). Note: In traditional offline trials, an open trial (open-label trial) is a type of clinical trial in which both the researchers and participants know which treatment is being administered. To avoid confusion, use “blinded” or “unblinded” to indicated the level of blinding instead of “open”, as “open” in web-based trials usually refers to “open access” (i.e. participants can self-enrol). (Note: Only report in the abstract what the main paper is reporting. If this information is missing from the main body of text, consider adding it)

|  |  |  |  |  |  |  |
| --- | --- | --- | --- | --- | --- | --- |
|  | 1 | 2 | 3 | 4 | 5 |  |
| subitem not at all important |  |  |  |  |  | essential |

This is a required question

Does your paper address subitem 1b-iii?

Copy and paste relevant sections from the manuscript abstract (include quotes in quotation marks "like this" to indicate direct quotes from your manuscript), or elaborate on this item by providing additional information not in the ms, or briefly explain why the item is not applicable/relevant for your study

Participants with comorbid MDD and DM (Type 1 or 2) were recruited online, and randomised to an iCBT program with therapist support provided by phone and/or email (n=42) or a treatment as usual control group (TAU, n=49). Outcomes were assessed through web-based self-report questionnaires, and the trial was web-based with no face-to-face components. 

This is a required question

1b-iv) RESULTS section in abstract must contain use data

Report number of participants enrolled/assessed in each group, the use/uptake of the intervention (e.g., attrition/adherence metrics, use over time, number of logins etc.), in addition to primary/secondary outcomes. (Note: Only report in the abstract what the main paper is reporting. If this information is missing from the main body of text, consider adding it)

|  |  |  |  |  |  |  |
| --- | --- | --- | --- | --- | --- | --- |
|  | 1 | 2 | 3 | 4 | 5 |  |
| subitem not at all important |  |  |  |  |  | essential |

This is a required question

Does your paper address subitem 1b-iv?

Copy and paste relevant sections from the manuscript abstract (include quotes in quotation marks "like this" to indicate direct quotes from your manuscript), or elaborate on this item by providing additional information not in the ms, or briefly explain why the item is not applicable/relevant for your study

Results: Twenty-seven participants (66%) completed the iCBT program. 

This is a required question

1b-v) CONCLUSIONS/DISCUSSION in abstract for negative trials

Conclusions/Discussions in abstract for negative trials: Discuss the primary outcome - if the trial is negative (primary outcome not changed), and the intervention was not used, discuss whether negative results are attributable to lack of uptake and discuss reasons. (Note: Only report in the abstract what the main paper is reporting. If this information is missing from the main body of text, consider adding it)

|  |  |  |  |  |  |  |
| --- | --- | --- | --- | --- | --- | --- |
|  | 1 | 2 | 3 | 4 | 5 |  |
| subitem not at all important |  |  |  |  |  | essential |

This is a required question

Does your paper address subitem 1b-v?

Copy and paste relevant sections from the manuscript abstract (include quotes in quotation marks "like this" to indicate direct quotes from your manuscript), or elaborate on this item by providing additional information not in the ms, or briefly explain why the item is not applicable/relevant for your study

We failed to find a difference between the iCBT program and the control group on measures of physical health status. See discussion below.
Despite finding significant improvements in mental health and wellbeing, the positive effects of this program did not appear to translate to improved physical health outcomes/wellbeing. We failed to find a difference at post-treatment on the physical wellbeing subscale of the SF-12, somatic symptom severity on the PHQ-15, and self-reported HbA1c levels, and there was no evidence of improvement in self-reported physical health outcomes between post-treatment and 3-months follow-up in the iCBT group. Although these results may be due to lack of power, these preliminary findings suggest that although iCBT for depression improved mental health outcomes, at least in the short-term, it did not improve physical health outcomes. The relatively short follow-up period of this study is likely to have precluded us from finding a positive effect on physical health outcomes and self-management behaviours, which may be only be observable at long-term follow-up [45]. 

This is a required question

## INTRODUCTION

## 2a) In INTRODUCTION: Scientific background and explanation of rationale

2a-i) Problem and the type of system/solution

Describe the problem and the type of system/solution that is object of the study: intended as stand-alone intervention vs. incorporated in broader health care program? Intended for a particular patient population? Goals of the intervention, e.g., being more cost-effective to other interventions, replace or complement other solutions? (Note: Details about the intervention are provided in “Methods” under 5)

|  |  |  |  |  |  |  |
| --- | --- | --- | --- | --- | --- | --- |
|  | 1 | 2 | 3 | 4 | 5 |  |
| subitem not at all important |  |  |  |  |  | essential |

This is a required question

Does your paper address subitem 2a-i?
\*

Copy and paste relevant sections from the manuscript (include quotes in quotation marks "like this" to indicate direct quotes from your manuscript), or elaborate on this item by providing additional information not in the ms, or briefly explain why the item is not applicable/relevant for your study

see text from manuscript:
Delivering evidence-based psychological treatments via the internet has potential to overcome some of these barriers. The efficacy of therapist-supported internet-delivered CBT (or iCBT) is now well-established for the treatment of depression and anxiety disorders in the general population [14], and these positive effects generalise to routine care [15]. Internet CBT programs are now successfully being used to encourage proactive self-management of a range of chronic health conditions including chronic pain and irritable bowel syndrome [16]. iCBT has been shown to be as efficacious as face-to-face CBT [17], but can be delivered at a fraction of the cost and clinical time [18]. In contrast to pharmacotherapies which often have significant side effects [19], iCBT does not lead to harm, and deterioration in symptoms is rare [20]. 

This is a required question

2a-ii) Scientific background, rationale: What is known about the (type of) system

Scientific background, rationale: What is known about the (type of) system that is the object of the study (be sure to discuss the use of similar systems for other conditions/diagnoses, if appropiate), motivation for the study, i.e. what are the reasons for and what is the context for this specific study, from which stakeholder viewpoint is the study performed, potential impact of findings [2]. Briefly justify the choice of the comparator.

|  |  |  |  |  |  |  |
| --- | --- | --- | --- | --- | --- | --- |
|  | 1 | 2 | 3 | 4 | 5 |  |
| subitem not at all important |  |  |  |  |  | essential |

This is a required question

Does your paper address subitem 2a-ii?
\*

Copy and paste relevant sections from the manuscript (include quotes in quotation marks "like this" to indicate direct quotes from your manuscript), or elaborate on this item by providing additional information not in the ms, or briefly explain why the item is not applicable/relevant for your study

Yes, see below.
These trials show promising results for using online CBT programs to treat depression symptoms in people with DM, although it is not known whether results generalise to clinically depressed individuals with MDD. To our knowledge, there are no studies examining whether general/unmodified iCBT depression programs - without any content tailored to DM - are effective for people with DM. With generic evidence-based iCBT programs for depression now available for general public use, they present a novel opportunity to reduce the problem of depression in routine DM care, but need to be tested first to evaluate whether they are acceptable and effective in people with DM. Although RCTs have shown that face-to-face protocol-driven CBT interventions are effective for treating depression in DM, in-person CBT differs to internet CBT in that it is able to be tailored and individualised to the individuals presenting problems. In contrast iCBT programs are typically highly standardised and fixed in format, and therefore need to be evaluated further in the context of DM. 

This is a required question

## 2b) In INTRODUCTION: Specific objectives or hypotheses

Does your paper address CONSORT subitem 2b?
\*

Copy and paste relevant sections from the manuscript (include quotes in quotation marks "like this" to indicate direct quotes from your manuscript), or elaborate on this item by providing additional information not in the ms, or briefly explain why the item is not applicable/relevant for your study

Yes, see below.
We hypothesised that adults with T1 or T2 DM who received iCBT would demonstrate significantly lower levels of depression, diabetes-related distress, and better self-reported glycaemic control compared to the TAU control group at post-treatment. 

This is a required question

## METHODS

## 3a) Description of trial design (such as parallel, factorial) including allocation ratio

Does your paper address CONSORT subitem 3a?
\*

Copy and paste relevant sections from the manuscript (include quotes in quotation marks "like this" to indicate direct quotes from your manuscript), or elaborate on this item by providing additional information not in the ms, or briefly explain why the item is not applicable/relevant for your study

Yes, see below.
Eligible participants were randomly allocated to the iCBT program or the TAU group who received the intervention after a 10-week waiting period. Simple randomisation was used (1:1 ratio), and randomisation numbers were generated by an independent research assistant using random.org. Group allocation was concealed in sequentially numbered opaque sealed envelopes. All participants provided electronic informed consent before participating.

This is a required question

## 3b) Important changes to methods after trial commencement (such as eligibility criteria), with reasons

Does your paper address CONSORT subitem 3b?
\*

Copy and paste relevant sections from the manuscript (include quotes in quotation marks "like this" to indicate direct quotes from your manuscript), or elaborate on this item by providing additional information not in the ms, or briefly explain why the item is not applicable/relevant for your study

N/A.

This is a required question

3b-i) Bug fixes, Downtimes, Content Changes

Bug fixes, Downtimes, Content Changes: ehealth systems are often dynamic systems. A description of changes to methods therefore also includes important changes made on the intervention or comparator during the trial (e.g., major bug fixes or changes in the functionality or content) (5-iii) and other “unexpected events” that may have influenced study design such as staff changes, system failures/downtimes, etc. [2].

|  |  |  |  |  |  |  |
| --- | --- | --- | --- | --- | --- | --- |
|  | 1 | 2 | 3 | 4 | 5 |  |
| subitem not at all important |  |  |  |  |  | essential |

This is a required question

Does your paper address subitem 3b-i?

Copy and paste relevant sections from the manuscript (include quotes in quotation marks "like this" to indicate direct quotes from your manuscript), or elaborate on this item by providing additional information not in the ms, or briefly explain why the item is not applicable/relevant for your study

N/A

This is a required question

## 4a) Eligibility criteria for participants

Does your paper address CONSORT subitem 4a?
\*

Copy and paste relevant sections from the manuscript (include quotes in quotation marks "like this" to indicate direct quotes from your manuscript), or elaborate on this item by providing additional information not in the ms, or briefly explain why the item is not applicable/relevant for your study

Yes, see below.,
Eligibility
Inclusion criteria included: 18 years or older, Australian resident, fluent in English, have access to a computer and internet, self-reported diagnosis of T1 or T2 DM, meet criteria for MDD according to telephone-administered diagnostic interview, and provide personal and general practitioner (GP) contact details. Participants were excluded if they had a self-reported diagnosis of bipolar affective disorder, psychotic disorder or substance use disorder, or were taking antipsychotics or benzodiazepines. Participants were excluded if they had commenced CBT in the past month, or changed antidepressant medication in the past two months. Participants scoring either <5 (normal range) or >23 (very severe) on the PHQ-9 were excluded, and those identified as being at significant risk of suicide or deliberate self-harm in the telephone risk assessment were also excluded and referred to appropriate treatments.

This is a required question

4a-i) Computer / Internet literacy

Computer / Internet literacy is often an implicit “de facto” eligibility criterion - this should be explicitly clarified.

|  |  |  |  |  |  |  |
| --- | --- | --- | --- | --- | --- | --- |
|  | 1 | 2 | 3 | 4 | 5 |  |
| subitem not at all important |  |  |  |  |  | essential |

This is a required question

Does your paper address subitem 4a-i?

Copy and paste relevant sections from the manuscript (include quotes in quotation marks "like this" to indicate direct quotes from your manuscript), or elaborate on this item by providing additional information not in the ms, or briefly explain why the item is not applicable/relevant for your study

We did not include this as an explicit inclusion criteria. 

This is a required question

4a-ii) Open vs. closed, web-based vs. face-to-face assessments:

Open vs. closed, web-based vs. face-to-face assessments: Mention how participants were recruited (online vs. offline), e.g., from an open access website or from a clinic, and clarify if this was a purely web-based trial, or there were face-to-face components (as part of the intervention or for assessment), i.e., to what degree got the study team to know the participant. In online-only trials, clarify if participants were quasi-anonymous and whether having multiple identities was possible or whether technical or logistical measures (e.g., cookies, email confirmation, phone calls) were used to detect/prevent these.

|  |  |  |  |  |  |  |
| --- | --- | --- | --- | --- | --- | --- |
|  | 1 | 2 | 3 | 4 | 5 |  |
| subitem not at all important |  |  |  |  |  | essential |

This is a required question

Does your paper address subitem 4a-ii?
\*

Copy and paste relevant sections from the manuscript (include quotes in quotation marks "like this" to indicate direct quotes from your manuscript), or elaborate on this item by providing additional information not in the ms, or briefly explain why the item is not applicable/relevant for your study

Participants were recruited between September 2013 to June 2015 by advertisements on DM websites, social media, and fliers. Interested applicants applied online via the Virtual Clinic website (www.virtualclinic.org.au), with an email and username, and completed online screening questionnaires. Those who passed the online screening criteria were assessed via telephone to confirm whether they met DSM-IV-TR diagnostic criteria [25] for MDD according to the MINI International Neuropsychiatric Interview Version 5.0.0 [26]. There were no face-to-face components of the study.

This is a required question

4a-iii) Information giving during recruitment

Information given during recruitment. Specify how participants were briefed for recruitment and in the informed consent procedures (e.g., publish the informed consent documentation as appendix, see also item X26), as this information may have an effect on user self-selection, user expectation and may also bias results.

|  |  |  |  |  |  |  |
| --- | --- | --- | --- | --- | --- | --- |
|  | 1 | 2 | 3 | 4 | 5 |  |
| subitem not at all important |  |  |  |  |  | essential |

This is a required question

Does your paper address subitem 4a-iii?

Copy and paste relevant sections from the manuscript (include quotes in quotation marks "like this" to indicate direct quotes from your manuscript), or elaborate on this item by providing additional information not in the ms, or briefly explain why the item is not applicable/relevant for your study

We have specifically stated that "All participants provided electronic informed consent before participating"
However, we have not included further details about the information statement as we do not believe this adds to the manuscript. 

This is a required question

## 4b) Settings and locations where the data were collected

Does your paper address CONSORT subitem 4b?
\*

Copy and paste relevant sections from the manuscript (include quotes in quotation marks "like this" to indicate direct quotes from your manuscript), or elaborate on this item by providing additional information not in the ms, or briefly explain why the item is not applicable/relevant for your study

Yes,
Interested applicants applied online via the Virtual Clinic website (www.virtualclinic.org.au), with an email and username, and completed online screening questionnaires. 

This is a required question

4b-i) Report if outcomes were (self-)assessed through online questionnaires

Clearly report if outcomes were (self-)assessed through online questionnaires (as common in web-based trials) or otherwise.

|  |  |  |  |  |  |  |
| --- | --- | --- | --- | --- | --- | --- |
|  | 1 | 2 | 3 | 4 | 5 |  |
| subitem not at all important |  |  |  |  |  | essential |

This is a required question

Does your paper address subitem 4b-i?
\*

Copy and paste relevant sections from the manuscript (include quotes in quotation marks "like this" to indicate direct quotes from your manuscript), or elaborate on this item by providing additional information not in the ms, or briefly explain why the item is not applicable/relevant for your study

Yes, in the measurements section:
All measures were web-based self-report questionnaires, with the exception of the MINI diagnostic interview which was administered by telephone.

This is a required question

4b-ii) Report how institutional affiliations are displayed

Report how institutional affiliations are displayed to potential participants [on ehealth media], as affiliations with prestigious hospitals or universities may affect volunteer rates, use, and reactions with regards to an intervention.(Not a required item – describe only if this may bias results)

|  |  |  |  |  |  |  |
| --- | --- | --- | --- | --- | --- | --- |
|  | 1 | 2 | 3 | 4 | 5 |  |
| subitem not at all important |  |  |  |  |  | essential |

This is a required question

Does your paper address subitem 4b-ii?

Copy and paste relevant sections from the manuscript (include quotes in quotation marks "like this" to indicate direct quotes from your manuscript), or elaborate on this item by providing additional information not in the ms, or briefly explain why the item is not applicable/relevant for your study

We have not included this information, as we do not believe it biased results. 

This is a required question

## 5) The interventions for each group with sufficient details to allow replication, including how and when they were actually administered

5-i) Mention names, credential, affiliations of the developers, sponsors, and owners

Mention names, credential, affiliations of the developers, sponsors, and owners [6] (if authors/evaluators are owners or developer of the software, this needs to be declared in a “Conflict of interest” section or mentioned elsewhere in the manuscript).

|  |  |  |  |  |  |  |
| --- | --- | --- | --- | --- | --- | --- |
|  | 1 | 2 | 3 | 4 | 5 |  |
| subitem not at all important |  |  |  |  |  | essential |

This is a required question

Does your paper address subitem 5-i?

Copy and paste relevant sections from the manuscript (include quotes in quotation marks "like this" to indicate direct quotes from your manuscript), or elaborate on this item by providing additional information not in the ms, or briefly explain why the item is not applicable/relevant for your study

N/A - as it is clear the program was administered through the Virtual Clinic website, so any interested person in replicating the study can contact the researchers via that website and replicate the website/design if needed. 

This is a required question

5-ii) Describe the history/development process

Describe the history/development process of the application and previous formative evaluations (e.g., focus groups, usability testing), as these will have an impact on adoption/use rates and help with interpreting results.

|  |  |  |  |  |  |  |
| --- | --- | --- | --- | --- | --- | --- |
|  | 1 | 2 | 3 | 4 | 5 |  |
| subitem not at all important |  |  |  |  |  | essential |

This is a required question

Does your paper address subitem 5-ii?

Copy and paste relevant sections from the manuscript (include quotes in quotation marks "like this" to indicate direct quotes from your manuscript), or elaborate on this item by providing additional information not in the ms, or briefly explain why the item is not applicable/relevant for your study

The program has been evaluated previously, with the results from previous user testing and evaluations in RCTs and effectiveness studies reported in the current paper. The current trial was an extension to explore the efficacy of this generic iCBT program within people who have comorbid diabetes and major depressive disorder, and so it was not necessary to elaborate on development/history process. 

This is a required question

5-iii) Revisions and updating

Revisions and updating. Clearly mention the date and/or version number of the application/intervention (and comparator, if applicable) evaluated, or describe whether the intervention underwent major changes during the evaluation process, or whether the development and/or content was “frozen” during the trial. Describe dynamic components such as news feeds or changing content which may have an impact on the replicability of the intervention (for unexpected events see item 3b).

|  |  |  |  |  |  |  |
| --- | --- | --- | --- | --- | --- | --- |
|  | 1 | 2 | 3 | 4 | 5 |  |
| subitem not at all important |  |  |  |  |  | essential |

This is a required question

Does your paper address subitem 5-iii?

Copy and paste relevant sections from the manuscript (include quotes in quotation marks "like this" to indicate direct quotes from your manuscript), or elaborate on this item by providing additional information not in the ms, or briefly explain why the item is not applicable/relevant for your study

This is not relevant, as the program has remained the same after it was initially developed. 

This is a required question

5-iv) Quality assurance methods

Provide information on quality assurance methods to ensure accuracy and quality of information provided [1], if applicable.

|  |  |  |  |  |  |  |
| --- | --- | --- | --- | --- | --- | --- |
|  | 1 | 2 | 3 | 4 | 5 |  |
| subitem not at all important |  |  |  |  |  | essential |

This is a required question

Does your paper address subitem 5-iv?

Copy and paste relevant sections from the manuscript (include quotes in quotation marks "like this" to indicate direct quotes from your manuscript), or elaborate on this item by providing additional information not in the ms, or briefly explain why the item is not applicable/relevant for your study

Not applicable. 

This is a required question

5-v) Ensure replicability by publishing the source code, and/or providing screenshots/screen-capture video, and/or providing flowcharts of the algorithms used

Ensure replicability by publishing the source code, and/or providing screenshots/screen-capture video, and/or providing flowcharts of the algorithms used. Replicability (i.e., other researchers should in principle be able to replicate the study) is a hallmark of scientific reporting.

|  |  |  |  |  |  |  |
| --- | --- | --- | --- | --- | --- | --- |
|  | 1 | 2 | 3 | 4 | 5 |  |
| subitem not at all important |  |  |  |  |  | essential |

This is a required question

Does your paper address subitem 5-v?

Copy and paste relevant sections from the manuscript (include quotes in quotation marks "like this" to indicate direct quotes from your manuscript), or elaborate on this item by providing additional information not in the ms, or briefly explain why the item is not applicable/relevant for your study

We have not published source code, but the user can contact the researchers for access to the program if needed. 

This is a required question

5-vi) Digital preservation

Digital preservation: Provide the URL of the application, but as the intervention is likely to change or disappear over the course of the years; also make sure the intervention is archived (Internet Archive, webcitation.org, and/or publishing the source code or screenshots/videos alongside the article). As pages behind login screens cannot be archived, consider creating demo pages which are accessible without login.

|  |  |  |  |  |  |  |
| --- | --- | --- | --- | --- | --- | --- |
|  | 1 | 2 | 3 | 4 | 5 |  |
| subitem not at all important |  |  |  |  |  | essential |

This is a required question

Does your paper address subitem 5-vi?

Copy and paste relevant sections from the manuscript (include quotes in quotation marks "like this" to indicate direct quotes from your manuscript), or elaborate on this item by providing additional information not in the ms, or briefly explain why the item is not applicable/relevant for your study

The Virtual Clinic is regularly maintained and has been for the past 10 years, via St Vincent's Hospital and the University of New South Wales. 

This is a required question

5-vii) Access

Access: Describe how participants accessed the application, in what setting/context, if they had to pay (or were paid) or not, whether they had to be a member of specific group. If known, describe how participants obtained “access to the platform and Internet” [1]. To ensure access for editors/reviewers/readers, consider to provide a “backdoor” login account or demo mode for reviewers/readers to explore the application (also important for archiving purposes, see vi).

|  |  |  |  |  |  |  |
| --- | --- | --- | --- | --- | --- | --- |
|  | 1 | 2 | 3 | 4 | 5 |  |
| subitem not at all important |  |  |  |  |  | essential |

This is a required question

Does your paper address subitem 5-vii?
\*

Copy and paste relevant sections from the manuscript (include quotes in quotation marks "like this" to indicate direct quotes from your manuscript), or elaborate on this item by providing additional information not in the ms, or briefly explain why the item is not applicable/relevant for your study

We have included the suggestion that:
The iCBT Program is described in detail elsewhere [24, 27], and a demo can be accessed by contacting the corresponding author.
Therefore there is the possibility of gaining a demo code if the reader would wish to look at the program.

This is a required question

5-viii) Mode of delivery, features/functionalities/components of the intervention and comparator, and the theoretical framework

Describe mode of delivery, features/functionalities/components of the intervention and comparator, and the theoretical framework [6] used to design them (instructional strategy [1], behaviour change techniques, persuasive features, etc., see e.g., [7, 8] for terminology). This includes an in-depth description of the content (including where it is coming from and who developed it) [1],” whether [and how] it is tailored to individual circumstances and allows users to track their progress and receive feedback” [6]. This also includes a description of communication delivery channels and – if computer-mediated communication is a component – whether communication was synchronous or asynchronous [6]. It also includes information on presentation strategies [1], including page design principles, average amount of text on pages, presence of hyperlinks to other resources, etc. [1].

|  |  |  |  |  |  |  |
| --- | --- | --- | --- | --- | --- | --- |
|  | 1 | 2 | 3 | 4 | 5 |  |
| subitem not at all important |  |  |  |  |  | essential |

This is a required question

Does your paper address subitem 5-viii?
\*

Copy and paste relevant sections from the manuscript (include quotes in quotation marks "like this" to indicate direct quotes from your manuscript), or elaborate on this item by providing additional information not in the ms, or briefly explain why the item is not applicable/relevant for your study

The course is described in detail in the methods:
The iCBT Program is described in detail elsewhere [24, 27], and a demo can be accessed by contacting the corresponding author. In brief, participants completed 6 automated cartoon-style online lessons teaching CBT skills (e.g., behavioural activation) over 10 weeks, with a minimum wait-time of 5 days between lessons. Participants downloaded a “homework” document which included practical assignments (e.g., thought monitoring) after each lesson, and had access to extra resources, frequently asked questions and recovery stories of former participants. Automated reminder emails were also sent to participants when lessons became available. Participants in the iCBT group were able to continue to receive usual care from their health services during the intervention period. 

This is a required question

5-ix) Describe use parameters

Describe use parameters (e.g., intended “doses” and optimal timing for use). Clarify what instructions or recommendations were given to the user, e.g., regarding timing, frequency, heaviness of use, if any, or was the intervention used ad libitum.

|  |  |  |  |  |  |  |
| --- | --- | --- | --- | --- | --- | --- |
|  | 1 | 2 | 3 | 4 | 5 |  |
| subitem not at all important |  |  |  |  |  | essential |

This is a required question

Does your paper address subitem 5-ix?

Copy and paste relevant sections from the manuscript (include quotes in quotation marks "like this" to indicate direct quotes from your manuscript), or elaborate on this item by providing additional information not in the ms, or briefly explain why the item is not applicable/relevant for your study

The program was able to be used ad lib.

This is a required question

5-x) Clarify the level of human involvement

Clarify the level of human involvement (care providers or health professionals, also technical assistance) in the e-intervention or as co-intervention (detail number and expertise of professionals involved, if any, as well as “type of assistance offered, the timing and frequency of the support, how it is initiated, and the medium by which the assistance is delivered”. It may be necessary to distinguish between the level of human involvement required for the trial, and the level of human involvement required for a routine application outside of a RCT setting (discuss under item 21 – generalizability).

|  |  |  |  |  |  |  |
| --- | --- | --- | --- | --- | --- | --- |
|  | 1 | 2 | 3 | 4 | 5 |  |
| subitem not at all important |  |  |  |  |  | essential |

This is a required question

Does your paper address subitem 5-x?

Copy and paste relevant sections from the manuscript (include quotes in quotation marks "like this" to indicate direct quotes from your manuscript), or elaborate on this item by providing additional information not in the ms, or briefly explain why the item is not applicable/relevant for your study

We have clarified the clinician guidance in the methods section, and the results are also presented for the time spent per participant.
Minimal clinician-assistance was provided to encourage adherence and engagement with the program by trained clinical psychologists with either Masters (LR) or PhD-level (JN) qualifications or psychiatry registrars (TM). Clinicians contacted the patient after lesson 1 and lesson 2 by email or phone to encourage progress. During the remainder of the program, clinician contact was made primarily by email, but if clinically indicated, or if patients’ K-10 and/or PHQ-9 scores deteriorated significantly, telephone contact was made by the clinician.

This is a required question

5-xi) Report any prompts/reminders used

Report any prompts/reminders used: Clarify if there were prompts (letters, emails, phone calls, SMS) to use the application, what triggered them, frequency etc. It may be necessary to distinguish between the level of prompts/reminders required for the trial, and the level of prompts/reminders for a routine application outside of a RCT setting (discuss under item 21 – generalizability).

|  |  |  |  |  |  |  |
| --- | --- | --- | --- | --- | --- | --- |
|  | 1 | 2 | 3 | 4 | 5 |  |
| subitem not at all important |  |  |  |  |  | essential |

This is a required question

Does your paper address subitem 5-xi?
\*

Copy and paste relevant sections from the manuscript (include quotes in quotation marks "like this" to indicate direct quotes from your manuscript), or elaborate on this item by providing additional information not in the ms, or briefly explain why the item is not applicable/relevant for your study

This information is described in the method section.
Minimal clinician-assistance was provided to encourage adherence and engagement with the program by trained clinical psychologists with either Masters (LR) or PhD-level (JN) qualifications or psychiatry registrars (TM). Clinicians contacted the patient after lesson 1 and lesson 2 by email or phone to encourage progress. During the remainder of the program, clinician contact was made primarily by email, but if clinically indicated, or if patients’ K-10 and/or PHQ-9 scores deteriorated significantly, telephone contact was made by the clinician.

This is a required question

5-xii) Describe any co-interventions (incl. training/support)

Describe any co-interventions (incl. training/support): Clearly state any interventions that are provided in addition to the targeted eHealth intervention, as ehealth intervention may not be designed as stand-alone intervention. This includes training sessions and support [1]. It may be necessary to distinguish between the level of training required for the trial, and the level of training for a routine application outside of a RCT setting (discuss under item 21 – generalizability.

|  |  |  |  |  |  |  |
| --- | --- | --- | --- | --- | --- | --- |
|  | 1 | 2 | 3 | 4 | 5 |  |
| subitem not at all important |  |  |  |  |  | essential |

This is a required question

Does your paper address subitem 5-xii?
\*

Copy and paste relevant sections from the manuscript (include quotes in quotation marks "like this" to indicate direct quotes from your manuscript), or elaborate on this item by providing additional information not in the ms, or briefly explain why the item is not applicable/relevant for your study

Not applicable. 

This is a required question

## 6a) Completely defined pre-specified primary and secondary outcome measures, including how and when they were assessed

Does your paper address CONSORT subitem 6a?
\*

Copy and paste relevant sections from the manuscript (include quotes in quotation marks "like this" to indicate direct quotes from your manuscript), or elaborate on this item by providing additional information not in the ms, or briefly explain why the item is not applicable/relevant for your study

Yes, this has been included.
Primary Outcome Measures
The primary outcome measure was the Patient Health Questionnaire-9 (PHQ-9) [32], which is a validated 9-item self-report measure of depression symptom severity over the past two weeks. The PHQ-9 contains items answered on a 4-point Likert scale; the total score ranges between 0 and 27 [33]. The PHQ-9 has been validated in diabetes samples [34].
Glycaemic control was measured via self-reported haemoglobin A1c values (HbA1c). Diabetes-related distress was measured using the Problem Areas in Diabetes (PAID) [35] questionnaire, a well-validated 20-item measure with a 5-point Likert scale; total scores are multiplied by 1.25 and range from 0 to 100 (with higher scores indicating greater emotional distress). The PAID has demonstrated sensitivity to change [36] and good internal and test-retest reliability [35].
Secondary Outcome Measures
The secondary outcome measures include the: Kessler 10-item Psychological Distress scale (K-10) [37] for psychological distress; Short Form 12-item (SF-12) scale to measure of mental wellbeing (SF-12 MCS) and physical wellbeing (SF-12 PCS) [38], the Generalized Anxiety Disorder 7-Item (GAD-7) [39] for anxiety severity; and the Patient Health Questionnaire – Physical symptoms module for somatic symptom severity (PHQ-15) [40].

This is a required question

6a-i) Online questionnaires: describe if they were validated for online use and apply CHERRIES items to describe how the questionnaires were designed/deployed

If outcomes were obtained through online questionnaires, describe if they were validated for online use and apply CHERRIES items to describe how the questionnaires were designed/deployed [9].

|  |  |  |  |  |  |  |
| --- | --- | --- | --- | --- | --- | --- |
|  | 1 | 2 | 3 | 4 | 5 |  |
| subitem not at all important |  |  |  |  |  | essential |

This is a required question

Does your paper address subitem 6a-i?

Copy and paste relevant sections from manuscript text

This information including data on reliabiltiy and validity is included in the methods section for each measure.

This is a required question

6a-ii) Describe whether and how “use” (including intensity of use/dosage) was defined/measured/monitored

Describe whether and how “use” (including intensity of use/dosage) was defined/measured/monitored (logins, logfile analysis, etc.). Use/adoption metrics are important process outcomes that should be reported in any ehealth trial.

|  |  |  |  |  |  |  |
| --- | --- | --- | --- | --- | --- | --- |
|  | 1 | 2 | 3 | 4 | 5 |  |
| subitem not at all important |  |  |  |  |  | essential |

This is a required question

Does your paper address subitem 6a-ii?

Copy and paste relevant sections from manuscript text

We did not measure use, because of the restrictions of the data gathered from the website. 

This is a required question

6a-iii) Describe whether, how, and when qualitative feedback from participants was obtained

Describe whether, how, and when qualitative feedback from participants was obtained (e.g., through emails, feedback forms, interviews, focus groups).

|  |  |  |  |  |  |  |
| --- | --- | --- | --- | --- | --- | --- |
|  | 1 | 2 | 3 | 4 | 5 |  |
| subitem not at all important |  |  |  |  |  | essential |

This is a required question

Does your paper address subitem 6a-iii?

Copy and paste relevant sections from manuscript text

Not applicable. All data is quantitative in this study. 

This is a required question

## 6b) Any changes to trial outcomes after the trial commenced, with reasons

Does your paper address CONSORT subitem 6b?
\*

Copy and paste relevant sections from the manuscript (include quotes in quotation marks "like this" to indicate direct quotes from your manuscript), or elaborate on this item by providing additional information not in the ms, or briefly explain why the item is not applicable/relevant for your study

N/A

This is a required question

## 7a) How sample size was determined

NPT: When applicable, details of whether and how the clustering by care provides or centers was addressed

7a-i) Describe whether and how expected attrition was taken into account when calculating the sample size

Describe whether and how expected attrition was taken into account when calculating the sample size.

|  |  |  |  |  |  |  |
| --- | --- | --- | --- | --- | --- | --- |
|  | 1 | 2 | 3 | 4 | 5 |  |
| subitem not at all important |  |  |  |  |  | essential |

This is a required question

Does your paper address subitem 7a-i?

Copy and paste relevant sections from manuscript title (include quotes in quotation marks "like this" to indicate direct quotes from your manuscript), or elaborate on this item by providing additional information not in the ms, or briefly explain why the item is not applicable/relevant for your study

Power calculations
With a sample size of 40 participants per group, the study was powered (0.8 power) to detect a medium between-group difference of 0.65 on the primary depression measure at post-treatment (α set at .05).

This is a required question

## 7b) When applicable, explanation of any interim analyses and stopping guidelines

Does your paper address CONSORT subitem 7b?
\*

Copy and paste relevant sections from the manuscript (include quotes in quotation marks "like this" to indicate direct quotes from your manuscript), or elaborate on this item by providing additional information not in the ms, or briefly explain why the item is not applicable/relevant for your study

No interim analyses were conducted.

This is a required question

## 8a) Method used to generate the random allocation sequence

NPT: When applicable, how care providers were allocated to each trial group

Does your paper address CONSORT subitem 8a?
\*

Copy and paste relevant sections from the manuscript (include quotes in quotation marks "like this" to indicate direct quotes from your manuscript), or elaborate on this item by providing additional information not in the ms, or briefly explain why the item is not applicable/relevant for your study

Yes this is included
Study details are reported in the published protocol [24]. Eligible participants were randomly allocated to the iCBT program or the TAU group who received the intervention after a 10-week waiting period. Simple randomisation was used (1:1 ratio), and randomisation numbers were generated by an independent research assistant using random.org. Group allocation was concealed in sequentially numbered opaque sealed envelopes. All participants provided electronic informed consent before participating.

This is a required question

## 8b) Type of randomisation; details of any restriction (such as blocking and block size)

Does your paper address CONSORT subitem 8b?
\*

Copy and paste relevant sections from the manuscript (include quotes in quotation marks "like this" to indicate direct quotes from your manuscript), or elaborate on this item by providing additional information not in the ms, or briefly explain why the item is not applicable/relevant for your study

Yes, this is included.
Study details are reported in the published protocol [24]. Eligible participants were randomly allocated to the iCBT program or the TAU group who received the intervention after a 10-week waiting period. Simple randomisation was used (1:1 ratio), and randomisation numbers were generated by an independent research assistant using random.org. Group allocation was concealed in sequentially numbered opaque sealed envelopes. All participants provided electronic informed consent before participating.

This is a required question

## 9) Mechanism used to implement the random allocation sequence (such as sequentially numbered containers), describing any steps taken to conceal the sequence until interventions were assigned

Does your paper address CONSORT subitem 9?
\*

Copy and paste relevant sections from the manuscript (include quotes in quotation marks "like this" to indicate direct quotes from your manuscript), or elaborate on this item by providing additional information not in the ms, or briefly explain why the item is not applicable/relevant for your study

Yes this is included.
Study details are reported in the published protocol [24]. Eligible participants were randomly allocated to the iCBT program or the TAU group who received the intervention after a 10-week waiting period. Simple randomisation was used (1:1 ratio), and randomisation numbers were generated by an independent research assistant using random.org. Group allocation was concealed in sequentially numbered opaque sealed envelopes. All participants provided electronic informed consent before participating.

This is a required question

## 10) Who generated the random allocation sequence, who enrolled participants, and who assigned participants to interventions

Does your paper address CONSORT subitem 10?
\*

Copy and paste relevant sections from the manuscript (include quotes in quotation marks "like this" to indicate direct quotes from your manuscript), or elaborate on this item by providing additional information not in the ms, or briefly explain why the item is not applicable/relevant for your study

Yes, this is included.
Study details are reported in the published protocol [24]. Eligible participants were randomly allocated to the iCBT program or the TAU group who received the intervention after a 10-week waiting period. Simple randomisation was used (1:1 ratio), and randomisation numbers were generated by an independent research assistant using random.org. Group allocation was concealed in sequentially numbered opaque sealed envelopes. All participants provided electronic informed consent before participating.

This is a required question

## 11a) If done, who was blinded after assignment to interventions (for example, participants, care providers, those assessing outcomes) and how

NPT: Whether or not administering co-interventions were blinded to group assignment

11a-i) Specify who was blinded, and who wasn’t

Specify who was blinded, and who wasn’t. Usually, in web-based trials it is not possible to blind the participants [1, 3] (this should be clearly acknowledged), but it may be possible to blind outcome assessors, those doing data analysis or those administering co-interventions (if any).

|  |  |  |  |  |  |  |
| --- | --- | --- | --- | --- | --- | --- |
|  | 1 | 2 | 3 | 4 | 5 |  |
| subitem not at all important |  |  |  |  |  | essential |

This is a required question

Does your paper address subitem 11a-i?
\*

Copy and paste relevant sections from the manuscript (include quotes in quotation marks "like this" to indicate direct quotes from your manuscript), or elaborate on this item by providing additional information not in the ms, or briefly explain why the item is not applicable/relevant for your study

We specifically stated that the interviews were not blinded.

This is a required question

11a-ii) Discuss e.g., whether participants knew which intervention was the “intervention of interest” and which one was the “comparator”

Informed consent procedures (4a-ii) can create biases and certain expectations - discuss e.g., whether participants knew which intervention was the “intervention of interest” and which one was the “comparator”.

|  |  |  |  |  |  |  |
| --- | --- | --- | --- | --- | --- | --- |
|  | 1 | 2 | 3 | 4 | 5 |  |
| subitem not at all important |  |  |  |  |  | essential |

This is a required question

Does your paper address subitem 11a-ii?

Copy and paste relevant sections from the manuscript (include quotes in quotation marks "like this" to indicate direct quotes from your manuscript), or elaborate on this item by providing additional information not in the ms, or briefly explain why the item is not applicable/relevant for your study

It is not possible to blind participants to a control condition, as they knew that they were not receiving a web based intervention.

This is a required question

## 11b) If relevant, description of the similarity of interventions

(this item is usually not relevant for ehealth trials as it refers to similarity of a placebo or sham intervention to a active medication/intervention)

Does your paper address CONSORT subitem 11b?
\*

Copy and paste relevant sections from the manuscript (include quotes in quotation marks "like this" to indicate direct quotes from your manuscript), or elaborate on this item by providing additional information not in the ms, or briefly explain why the item is not applicable/relevant for your study

N/A

This is a required question

## 12a) Statistical methods used to compare groups for primary and secondary outcomes

NPT: When applicable, details of whether and how the clustering by care providers or centers was addressed

Does your paper address CONSORT subitem 12a?
\*

Copy and paste relevant sections from the manuscript (include quotes in quotation marks "like this" to indicate direct quotes from your manuscript), or elaborate on this item by providing additional information not in the ms, or briefly explain why the item is not applicable/relevant for your study

Yes,
Statistical analyses
All analyses were undertaken in SPSS v 23. Intention-to-treat (ITT) linear mixed models analyses were used to account for missing data due to participant drop-outs. This approach is appropriate for RCTs with multiple time points [28], and does not assume that the last measurement was stable [an assumption of the the last observation carried forward approach, 29]. Linear mixed models were conducted separately for each of the dependent variable (DV) measures, with time, treatment group, and the time by group interaction entered as fixed factors in the model, with a random intercept for subject. For each outcome, an identity covariance structure was specified to model the covariance structure of the random intercept. Initial model building focused on the selection of the most appropriate covariance structure for the residual correlation matrix. Model fit indices and inspection of the variance-covariance matrix supported the selection of the identity covariance structure for each of the outcome measures. The fixed effect of age was added to each of the models. For each outcome measure except for PAID scores, the fixed effect of age was not statistically significant and was removed from the model. Chi-square difference testing of the -2 log-likelihoods indicated that the removal of these fixed effects did not decrease model fit for any of the outcome variables, and they were excluded from further analyses.
For each group, planned contrasts were used to compare changes within and between-groups from baseline to post-treatment (and 3-month follow-up for the iCBT group only). Between-group effect sizes using the pooled standard deviation and adjusted for sample size (Hedges g) were calculated to compare between groups at post-treatment. Within-group effect sizes (Cohen’s d) were calculated between pre- and post-treatment for both groups, and between pre- and 3-month follow-up for the iCBT group only. Effect sizes of 0.2, 0.5 and 0.8 were considered to be small, moderate, and large respectively in line with Cohen’s recommendations [30]. Linear mixed models were also conducted with time entered as a fixed factor, and subject as a random intercept, for each DV to investigate whether there were changes between post-treatment and follow-up for the iCBT group (n=21).

This is a required question

12a-i) Imputation techniques to deal with attrition / missing values

Imputation techniques to deal with attrition / missing values: Not all participants will use the intervention/comparator as intended and attrition is typically high in ehealth trials. Specify how participants who did not use the application or dropped out from the trial were treated in the statistical analysis (a complete case analysis is strongly discouraged, and simple imputation techniques such as LOCF may also be problematic [4]).

|  |  |  |  |  |  |  |
| --- | --- | --- | --- | --- | --- | --- |
|  | 1 | 2 | 3 | 4 | 5 |  |
| subitem not at all important |  |  |  |  |  | essential |

This is a required question

Does your paper address subitem 12a-i?
\*

Copy and paste relevant sections from the manuscript (include quotes in quotation marks "like this" to indicate direct quotes from your manuscript), or elaborate on this item by providing additional information not in the ms, or briefly explain why the item is not applicable/relevant for your study

We used linear mixed models which account for missing data due to drop-outs, and is superior to LOCF analyses.
treatment (α set at .05).
Statistical analyses
All analyses were undertaken in SPSS v 23. Intention-to-treat (ITT) linear mixed models analyses were used to account for missing data due to participant drop-outs. This approach is appropriate for RCTs with multiple time points [28], and does not assume that the last measurement was stable [an assumption of the the last observation carried forward approach, 29]. Linear mixed models were conducted separately for each of the dependent variable (DV) measures, with time, treatment group, and the time by group interaction entered as fixed factors in the model, with a random intercept for subject. For each outcome, an identity covariance structure was specified to model the covariance structure of the random intercept. Initial model building focused on the selection of the most appropriate covariance structure for the residual correlation matrix. Model fit indices and inspection of the variance-covariance matrix supported the selection of the identity covariance structure for each of the outcome measures. The fixed effect of age was added to each of the models. For each outcome measure except for PAID scores, the fixed effect of age was not statistically significant and was removed from the model. Chi-square difference testing of the -2 log-likelihoods indicated that the removal of these fixed effects did not decrease model fit for any of the outcome variables, and they were excluded from further analyses.
For each group, planned contrasts were used to compare changes within and between-groups from baseline to post-treatment (and 3-month follow-up for the iCBT group only). Between-group effect sizes using the pooled standard deviation and adjusted for sample size (Hedges g) were calculated to compare between groups at post-treatment. Within-group effect sizes (Cohen’s d) were calculated between pre- and post-treatment for both groups, and between pre- and 3-month follow-up for the iCBT group only. Effect sizes of 0.2, 0.5 and 0.8 were considered to be small, moderate, and large respectively in line with Cohen’s recommendations [30]. Linear mixed models were also conducted with time entered as a fixed factor, and subject as a random intercept, for each DV to investigate whether there were changes between post-treatment and follow-up for the iCBT group (n=21).

This is a required question

## 12b) Methods for additional analyses, such as subgroup analyses and adjusted analyses

Does your paper address CONSORT subitem 12b?
\*

Copy and paste relevant sections from the manuscript (include quotes in quotation marks "like this" to indicate direct quotes from your manuscript), or elaborate on this item by providing additional information not in the ms, or briefly explain why the item is not applicable/relevant for your study

N/A

This is a required question

## X26) REB/IRB Approval and Ethical Considerations [recommended as subheading under "Methods"] (not a CONSORT item)

X26-i) Comment on ethics committee approval

|  |  |  |  |  |  |  |
| --- | --- | --- | --- | --- | --- | --- |
|  | 1 | 2 | 3 | 4 | 5 |  |
| subitem not at all important |  |  |  |  |  | essential |

This is a required question

Does your paper address subitem X26-i?

Copy and paste relevant sections from the manuscript (include quotes in quotation marks "like this" to indicate direct quotes from your manuscript), or elaborate on this item by providing additional information not in the ms, or briefly explain why the item is not applicable/relevant for your study

This study was approved by the St Vincent’s Hospital Human Research Ethics Committee (HREC/13/SVH/291). The trial was registered with the Australian and New Zealand Clinical Trials registry (ACTRN12613001198718). 

This is a required question

x26-ii) Outline informed consent procedures

Outline informed consent procedures e.g., if consent was obtained offline or online (how? Checkbox, etc.?), and what information was provided (see 4a-ii). See [6] for some items to be included in informed consent documents.

|  |  |  |  |  |  |  |
| --- | --- | --- | --- | --- | --- | --- |
|  | 1 | 2 | 3 | 4 | 5 |  |
| subitem not at all important |  |  |  |  |  | essential |

This is a required question

Does your paper address subitem X26-ii?

Copy and paste relevant sections from the manuscript (include quotes in quotation marks "like this" to indicate direct quotes from your manuscript), or elaborate on this item by providing additional information not in the ms, or briefly explain why the item is not applicable/relevant for your study

 All participants provided electronic informed consent before participating.

This is a required question

X26-iii) Safety and security procedures

Safety and security procedures, incl. privacy considerations, and any steps taken to reduce the likelihood or detection of harm (e.g., education and training, availability of a hotline)

|  |  |  |  |  |  |  |
| --- | --- | --- | --- | --- | --- | --- |
|  | 1 | 2 | 3 | 4 | 5 |  |
| subitem not at all important |  |  |  |  |  | essential |

This is a required question

Does your paper address subitem X26-iii?

Copy and paste relevant sections from the manuscript (include quotes in quotation marks "like this" to indicate direct quotes from your manuscript), or elaborate on this item by providing additional information not in the ms, or briefly explain why the item is not applicable/relevant for your study

Not applicable.

This is a required question

## RESULTS

## 13a) For each group, the numbers of participants who were randomly assigned, received intended treatment, and were analysed for the primary outcome

NPT: The number of care providers or centers performing the intervention in each group and the number of patients treated by each care provider in each center

Does your paper address CONSORT subitem 13a?
\*

Copy and paste relevant sections from the manuscript (include quotes in quotation marks "like this" to indicate direct quotes from your manuscript), or elaborate on this item by providing additional information not in the ms, or briefly explain why the item is not applicable/relevant for your study

Yes this is included in the results.
Participant Flow
Of the 334 individuals who started an online application, 185 were eligible for phone interview. After phone interview, 106 individuals met inclusion criteria and were randomised to either iCBT (n=49), or TAU (n=57). Of these participants, 42/49 allocated to the iCBT group and 49/57 allocated to the TAU group completed baseline assessment and were included in the Intention to Treat (ITT) analysis. At post-treatment assessment, 31/49 provided data in the iCBT group, and 46/49 provided data in the TAU group. At 3-month follow-up, 21 participants completed the questionnaires, and 31 completed 

This is a required question

## 13b) For each group, losses and exclusions after randomisation, together with reasons

Does your paper address CONSORT subitem 13b? (NOTE: Preferably, this is shown in a CONSORT flow diagram)
\*

Copy and paste relevant sections from the manuscript (include quotes in quotation marks "like this" to indicate direct quotes from your manuscript), or elaborate on this item by providing additional information not in the ms, or briefly explain why the item is not applicable/relevant for your study

Yes all of the information is in the participant flow and flow diagram..
Participant Flow
Of the 334 individuals who started an online application, 185 were eligible for phone interview. After phone interview, 106 individuals met inclusion criteria and were randomised to either iCBT (n=49), or TAU (n=57). Of these participants, 42/49 allocated to the iCBT group and 49/57 allocated to the TAU group completed baseline assessment and were included in the Intention to Treat (ITT) analysis. At post-treatment assessment, 31/49 provided data in the iCBT group, and 46/49 provided data in the TAU group. At 3-month follow-up, 21 participants completed the questionnaires, and 31 completed 

This is a required question

13b-i) Attrition diagram

Strongly recommended: An attrition diagram (e.g., proportion of participants still logging in or using the intervention/comparator in each group plotted over time, similar to a survival curve) or other figures or tables demonstrating usage/dose/engagement.

|  |  |  |  |  |  |  |
| --- | --- | --- | --- | --- | --- | --- |
|  | 1 | 2 | 3 | 4 | 5 |  |
| subitem not at all important |  |  |  |  |  | essential |

This is a required question

Does your paper address subitem 13b-i?

Copy and paste relevant sections from the manuscript or cite the figure number if applicable (include quotes in quotation marks "like this" to indicate direct quotes from your manuscript), or elaborate on this item by providing additional information not in the ms, or briefly explain why the item is not applicable/relevant for your study

Yes this is included, see page 13

This is a required question

## 14a) Dates defining the periods of recruitment and follow-up

Does your paper address CONSORT subitem 14a?
\*

Copy and paste relevant sections from the manuscript (include quotes in quotation marks "like this" to indicate direct quotes from your manuscript), or elaborate on this item by providing additional information not in the ms, or briefly explain why the item is not applicable/relevant for your study

Yes this is included on page. 8
Participants were recruited between September 2013 to June 2015 by advertisements on DM websites, social media, and fliers. 

This is a required question

14a-i) Indicate if critical “secular events” fell into the study period

Indicate if critical “secular events” fell into the study period, e.g., significant changes in Internet resources available or “changes in computer hardware or Internet delivery resources”

|  |  |  |  |  |  |  |
| --- | --- | --- | --- | --- | --- | --- |
|  | 1 | 2 | 3 | 4 | 5 |  |
| subitem not at all important |  |  |  |  |  | essential |

This is a required question

Does your paper address subitem 14a-i?

Copy and paste relevant sections from the manuscript (include quotes in quotation marks "like this" to indicate direct quotes from your manuscript), or elaborate on this item by providing additional information not in the ms, or briefly explain why the item is not applicable/relevant for your study

Not applicable.

This is a required question

## 14b) Why the trial ended or was stopped (early)

Does your paper address CONSORT subitem 14b?
\*

Copy and paste relevant sections from the manuscript (include quotes in quotation marks "like this" to indicate direct quotes from your manuscript), or elaborate on this item by providing additional information not in the ms, or briefly explain why the item is not applicable/relevant for your study

N/A

This is a required question

## 15) A table showing baseline demographic and clinical characteristics for each group

NPT: When applicable, a description of care providers (case volume, qualification, expertise, etc.) and centers (volume) in each group

Does your paper address CONSORT subitem 15?
\*

Copy and paste relevant sections from the manuscript (include quotes in quotation marks "like this" to indicate direct quotes from your manuscript), or elaborate on this item by providing additional information not in the ms, or briefly explain why the item is not applicable/relevant for your study

Yes, see table 1 with baseline characteristics.

This is a required question

15-i) Report demographics associated with digital divide issues

In ehealth trials it is particularly important to report demographics associated with digital divide issues, such as age, education, gender, social-economic status, computer/Internet/ehealth literacy of the participants, if known.

|  |  |  |  |  |  |  |
| --- | --- | --- | --- | --- | --- | --- |
|  | 1 | 2 | 3 | 4 | 5 |  |
| subitem not at all important |  |  |  |  |  | essential |

This is a required question

Does your paper address subitem 15-i?
\*

Copy and paste relevant sections from the manuscript (include quotes in quotation marks "like this" to indicate direct quotes from your manuscript), or elaborate on this item by providing additional information not in the ms, or briefly explain why the item is not applicable/relevant for your study

Yes, we have included age, education, gender. Not the other variables. see Table 1 for information, and description in the results section.
Fifty-two participants (57% of the sample) had T1 DM. Participants were 47 years on average (SD=12.61, range=20-71), and the majority were female (71.1%, n=64), and married or living in a de-facto relationship (n=55, 61.1%). Education status was mixed: one fifth (n=16, 17.8%) had not completed high school, whereas 29 completed tertiary education (32.2%). The majority were in full-time or part-time paid work (n=50, 55.6%), with 12 on the disability support pension (5.6%). Only 16% (n=14) of the total sample were receiving psychological therapy, and 40% were taking medications for depression at baseline (n=37, 41.1%; see Table 1 for sample characteristics). Participants’ baseline depression levels were moderate to severe on the PHQ-9 (M=15.0, SD=5.3). The majority reported 3 or more episodes of depression (n=80, 88.9%); two thirds of the sample had not been depression free for at least 2 years (n=57, 63.3%), and half (n=46, 51.1%) reported being depressed for more than 4 years during their lifetime. 

This is a required question

## 16) For each group, number of participants (denominator) included in each analysis and whether the analysis was by original assigned groups

16-i) Report multiple “denominators” and provide definitions

Report multiple “denominators” and provide definitions: Report N’s (and effect sizes) “across a range of study participation [and use] thresholds” [1], e.g., N exposed, N consented, N used more than x times, N used more than y weeks, N participants “used” the intervention/comparator at specific pre-defined time points of interest (in absolute and relative numbers per group). Always clearly define “use” of the intervention.

|  |  |  |  |  |  |  |
| --- | --- | --- | --- | --- | --- | --- |
|  | 1 | 2 | 3 | 4 | 5 |  |
| subitem not at all important |  |  |  |  |  | essential |

This is a required question

Does your paper address subitem 16-i?
\*

Copy and paste relevant sections from the manuscript (include quotes in quotation marks "like this" to indicate direct quotes from your manuscript), or elaborate on this item by providing additional information not in the ms, or briefly explain why the item is not applicable/relevant for your study

Yes this included in the tables and the outcomes reports. 

This is a required question

16-ii) Primary analysis should be intent-to-treat

Primary analysis should be intent-to-treat, secondary analyses could include comparing only “users”, with the appropriate caveats that this is no longer a randomized sample (see 18-i).

|  |  |  |  |  |  |  |
| --- | --- | --- | --- | --- | --- | --- |
|  | 1 | 2 | 3 | 4 | 5 |  |
| subitem not at all important |  |  |  |  |  | essential |

This is a required question

Does your paper address subitem 16-ii?

Copy and paste relevant sections from the manuscript (include quotes in quotation marks "like this" to indicate direct quotes from your manuscript), or elaborate on this item by providing additional information not in the ms, or briefly explain why the item is not applicable/relevant for your study

Yes we have specifically mentioned this, see above. 

This is a required question

## 17a) For each primary and secondary outcome, results for each group, and the estimated effect size and its precision (such as 95% confidence interval)

Does your paper address CONSORT subitem 17a?
\*

Copy and paste relevant sections from the manuscript (include quotes in quotation marks "like this" to indicate direct quotes from your manuscript), or elaborate on this item by providing additional information not in the ms, or briefly explain why the item is not applicable/relevant for your study

Yes, these are included throughout the manuscript including the tables reporting the final results. 

This is a required question

17a-i) Presentation of process outcomes such as metrics of use and intensity of use

In addition to primary/secondary (clinical) outcomes, the presentation of process outcomes such as metrics of use and intensity of use (dose, exposure) and their operational definitions is critical. This does not only refer to metrics of attrition (13-b) (often a binary variable), but also to more continuous exposure metrics such as “average session length”. These must be accompanied by a technical description how a metric like a “session” is defined (e.g., timeout after idle time) [1] (report under item 6a).

|  |  |  |  |  |  |  |
| --- | --- | --- | --- | --- | --- | --- |
|  | 1 | 2 | 3 | 4 | 5 |  |
| subitem not at all important |  |  |  |  |  | essential |

This is a required question

Does your paper address subitem 17a-i?

Copy and paste relevant sections from the manuscript (include quotes in quotation marks "like this" to indicate direct quotes from your manuscript), or elaborate on this item by providing additional information not in the ms, or briefly explain why the item is not applicable/relevant for your study

N/A we only have the frequency of clinician contact, not the frequency of use as we were unable to extract that from the website. 

This is a required question

## 17b) For binary outcomes, presentation of both absolute and relative effect sizes is recommended

Does your paper address CONSORT subitem 17b?
\*

Copy and paste relevant sections from the manuscript (include quotes in quotation marks "like this" to indicate direct quotes from your manuscript), or elaborate on this item by providing additional information not in the ms, or briefly explain why the item is not applicable/relevant for your study

N./A - all of our measures are dimensional.

This is a required question

## 18) Results of any other analyses performed, including subgroup analyses and adjusted analyses, distinguishing pre-specified from exploratory

Does your paper address CONSORT subitem 18?
\*

Copy and paste relevant sections from the manuscript (include quotes in quotation marks "like this" to indicate direct quotes from your manuscript), or elaborate on this item by providing additional information not in the ms, or briefly explain why the item is not applicable/relevant for your study

N/A - all were a priori analyses.

This is a required question

18-i) Subgroup analysis of comparing only users

A subgroup analysis of comparing only users is not uncommon in ehealth trials, but if done, it must be stressed that this is a self-selected sample and no longer an unbiased sample from a randomized trial (see 16-iii).

|  |  |  |  |  |  |  |
| --- | --- | --- | --- | --- | --- | --- |
|  | 1 | 2 | 3 | 4 | 5 |  |
| subitem not at all important |  |  |  |  |  | essential |

This is a required question

Does your paper address subitem 18-i?

Copy and paste relevant sections from the manuscript (include quotes in quotation marks "like this" to indicate direct quotes from your manuscript), or elaborate on this item by providing additional information not in the ms, or briefly explain why the item is not applicable/relevant for your study

Not applicable. 

This is a required question

## 19) All important harms or unintended effects in each group

(for specific guidance see CONSORT for harms)

Does your paper address CONSORT subitem 19?
\*

Copy and paste relevant sections from the manuscript (include quotes in quotation marks "like this" to indicate direct quotes from your manuscript), or elaborate on this item by providing additional information not in the ms, or briefly explain why the item is not applicable/relevant for your study

We did not collect this data. 

This is a required question

19-i) Include privacy breaches, technical problems

Include privacy breaches, technical problems. This does not only include physical “harm” to participants, but also incidents such as perceived or real privacy breaches [1], technical problems, and other unexpected/unintended incidents. “Unintended effects” also includes unintended positive effects [2].

|  |  |  |  |  |  |  |
| --- | --- | --- | --- | --- | --- | --- |
|  | 1 | 2 | 3 | 4 | 5 |  |
| subitem not at all important |  |  |  |  |  | essential |

This is a required question

Does your paper address subitem 19-i?

Copy and paste relevant sections from the manuscript (include quotes in quotation marks "like this" to indicate direct quotes from your manuscript), or elaborate on this item by providing additional information not in the ms, or briefly explain why the item is not applicable/relevant for your study

N/A

This is a required question

19-ii) Include qualitative feedback from participants or observations from staff/researchers

Include qualitative feedback from participants or observations from staff/researchers, if available, on strengths and shortcomings of the application, especially if they point to unintended/unexpected effects or uses. This includes (if available) reasons for why people did or did not use the application as intended by the developers.

|  |  |  |  |  |  |  |
| --- | --- | --- | --- | --- | --- | --- |
|  | 1 | 2 | 3 | 4 | 5 |  |
| subitem not at all important |  |  |  |  |  | essential |

This is a required question

Does your paper address subitem 19-ii?

Copy and paste relevant sections from the manuscript (include quotes in quotation marks "like this" to indicate direct quotes from your manuscript), or elaborate on this item by providing additional information not in the ms, or briefly explain why the item is not applicable/relevant for your study

Not applicable - we did not collect qualitative data for this study. 

This is a required question

## DISCUSSION

## 22) Interpretation consistent with results, balancing benefits and harms, and considering other relevant evidence

NPT: In addition, take into account the choice of the comparator, lack of or partial blinding, and unequal expertise of care providers or centers in each group

22-i) Restate study questions and summarize the answers suggested by the data, starting with primary outcomes and process outcomes (use)

Restate study questions and summarize the answers suggested by the data, starting with primary outcomes and process outcomes (use).

|  |  |  |  |  |  |  |
| --- | --- | --- | --- | --- | --- | --- |
|  | 1 | 2 | 3 | 4 | 5 |  |
| subitem not at all important |  |  |  |  |  | essential |

This is a required question

Does your paper address subitem 22-i?
\*

Copy and paste relevant sections from the manuscript (include quotes in quotation marks "like this" to indicate direct quotes from your manuscript), or elaborate on this item by providing additional information not in the ms, or briefly explain why the item is not applicable/relevant for your study

See entire discussion section, where we discuss results, data and implications. 

This is a required question

22-ii) Highlight unanswered new questions, suggest future research

Highlight unanswered new questions, suggest future research.

|  |  |  |  |  |  |  |
| --- | --- | --- | --- | --- | --- | --- |
|  | 1 | 2 | 3 | 4 | 5 |  |
| subitem not at all important |  |  |  |  |  | essential |

This is a required question

Does your paper address subitem 22-ii?

Copy and paste relevant sections from the manuscript (include quotes in quotation marks "like this" to indicate direct quotes from your manuscript), or elaborate on this item by providing additional information not in the ms, or briefly explain why the item is not applicable/relevant for your study

We include many unanswered new questions and future research suggestions throughout the discussion. 

This is a required question

## 20) Trial limitations, addressing sources of potential bias, imprecision, and, if relevant, multiplicity of analyses

20-i) Typical limitations in ehealth trials

Typical limitations in ehealth trials: Participants in ehealth trials are rarely blinded. Ehealth trials often look at a multiplicity of outcomes, increasing risk for a Type I error. Discuss biases due to non-use of the intervention/usability issues, biases through informed consent procedures, unexpected events.

|  |  |  |  |  |  |  |
| --- | --- | --- | --- | --- | --- | --- |
|  | 1 | 2 | 3 | 4 | 5 |  |
| subitem not at all important |  |  |  |  |  | essential |

This is a required question

Does your paper address subitem 20-i?
\*

Copy and paste relevant sections from the manuscript (include quotes in quotation marks "like this" to indicate direct quotes from your manuscript), or elaborate on this item by providing additional information not in the ms, or briefly explain why the item is not applicable/relevant for your study

Yes, we have included a limitations section
Limitations
The findings should be interpreted in the context of its limitations. Because we do not have follow-up data in the TAU group, more research is needed to confirm the efficacy of iCBT for depression in people with DM in the long-term (e.g., 6-12 months following treatment). Follow-up diagnostic interviews were not blinded to treatment allocation, introducing bias. The use of self-reported HbA1c levels to assess glycaemic control was also a limitation of the study, and we did not assess when the participants had their HbA1c levels tested. We chose this measure to mirror what occurs in usual clinical care, because we wanted to increase the coverage of recruitment across Australia, and it was impractical to collect blood samples in other Australian states and territories. Finally, the high proportion of females and people with T1 DM may influence the generalisability of the findings

This is a required question

## 21) Generalisability (external validity, applicability) of the trial findings

NPT: External validity of the trial findings according to the intervention, comparators, patients, and care providers or centers involved in the trial

21-i) Generalizability to other populations

Generalizability to other populations: In particular, discuss generalizability to a general Internet population, outside of a RCT setting, and general patient population, including applicability of the study results for other organizations

|  |  |  |  |  |  |  |
| --- | --- | --- | --- | --- | --- | --- |
|  | 1 | 2 | 3 | 4 | 5 |  |
| subitem not at all important |  |  |  |  |  | essential |

This is a required question

Does your paper address subitem 21-i?

Copy and paste relevant sections from the manuscript (include quotes in quotation marks "like this" to indicate direct quotes from your manuscript), or elaborate on this item by providing additional information not in the ms, or briefly explain why the item is not applicable/relevant for your study

We have discussed generalisability of the findings in the limitations section:
Limitations
The findings should be interpreted in the context of its limitations. Because we do not have follow-up data in the TAU group, more research is needed to confirm the efficacy of iCBT for depression in people with DM in the long-term (e.g., 6-12 months following treatment). Follow-up diagnostic interviews were not blinded to treatment allocation, introducing bias. The use of self-reported HbA1c levels to assess glycaemic control was also a limitation of the study, and we did not assess when the participants had their HbA1c levels tested. We chose this measure to mirror what occurs in usual clinical care, because we wanted to increase the coverage of recruitment across Australia, and it was impractical to collect blood samples in other Australian states and territories. Finally, the high proportion of females and people with T1 DM may influence the generalisability of the findings

This is a required question

21-ii) Discuss if there were elements in the RCT that would be different in a routine application setting

Discuss if there were elements in the RCT that would be different in a routine application setting (e.g., prompts/reminders, more human involvement, training sessions or other co-interventions) and what impact the omission of these elements could have on use, adoption, or outcomes if the intervention is applied outside of a RCT setting.

|  |  |  |  |  |  |  |
| --- | --- | --- | --- | --- | --- | --- |
|  | 1 | 2 | 3 | 4 | 5 |  |
| subitem not at all important |  |  |  |  |  | essential |

This is a required question

Does your paper address subitem 21-ii?

Copy and paste relevant sections from the manuscript (include quotes in quotation marks "like this" to indicate direct quotes from your manuscript), or elaborate on this item by providing additional information not in the ms, or briefly explain why the item is not applicable/relevant for your study

We have discussed the use of modified programs that tailor the content of the program for diabetes patients in routine care. Otherwise, the program is available to be used in routine care already. 

This is a required question

## OTHER INFORMATION

## 23) Registration number and name of trial registry

Does your paper address CONSORT subitem 23?
\*

Copy and paste relevant sections from the manuscript (include quotes in quotation marks "like this" to indicate direct quotes from your manuscript), or elaborate on this item by providing additional information not in the ms, or briefly explain why the item is not applicable/relevant for your study

Yes, see above.
Registration: Australian and New Zealand Clinical Trials Registry, ACTRN12613001198718.

This is a required question

## 24) Where the full trial protocol can be accessed, if available

Does your paper address CONSORT subitem 24?
\*

Cite a Multimedia Appendix, other reference, or copy and paste relevant sections from the manuscript (include quotes in quotation marks "like this" to indicate direct quotes from your manuscript), or elaborate on this item by providing additional information not in the ms, or briefly explain why the item is not applicable/relevant for your study

Yes, we have the trial protocol published. 

This is a required question

## 25) Sources of funding and other support (such as supply of drugs), role of funders

Does your paper address CONSORT subitem 25?
\*

Copy and paste relevant sections from the manuscript (include quotes in quotation marks "like this" to indicate direct quotes from your manuscript), or elaborate on this item by providing additional information not in the ms, or briefly explain why the item is not applicable/relevant for your study

Yes, we have included this information.
This research received no specific grant from any funding agency in the public, commercial or not-for-profit sectors. Dr Newby is supported by an Australian NHMRC Early Career Australian Clinical Fellowship (#1037797). Lisa Robins is supported by a grant from the St Vincent’s Clinic Foundation and an APA scholarship.

This is a required question

## X27) Conflicts of Interest (not a CONSORT item)

X27-i) State the relation of the study team towards the system being evaluated

In addition to the usual declaration of interests (financial or otherwise), also state the relation of the study team towards the system being evaluated, i.e., state if the authors/evaluators are distinct from or identical with the developers/sponsors of the intervention.

|  |  |  |  |  |  |  |
| --- | --- | --- | --- | --- | --- | --- |
|  | 1 | 2 | 3 | 4 | 5 |  |
| subitem not at all important |  |  |  |  |  | essential |

This is a required question

Does your paper address subitem X27-i?

Copy and paste relevant sections from the manuscript (include quotes in quotation marks "like this" to indicate direct quotes from your manuscript), or elaborate on this item by providing additional information not in the ms, or briefly explain why the item is not applicable/relevant for your study

Yes, there are no conflict of interest.
Conflicts of interest
All authors declare that they have no conflicts of interest.

This is a required question

## About the CONSORT EHEALTH checklist

As a result of using this checklist, did you make changes in your manuscript?
\*

- yes, major changes
- yes, minor changes
- no

This is a required question

What were the most important changes you made as a result of using this checklist?


This is a required question

How much time did you spend on going through the checklist INCLUDING making changes in your manuscript
\*

At least one hour. 

This is a required question

As a result of using this checklist, do you think your manuscript has improved?
\*

- yes
- no
- Other:

This is a required question

Would you like to become involved in the CONSORT EHEALTH group?

This would involve for example becoming involved in participating in a workshop and writing an "Explanation and Elaboration" document

- yes
- no
- Other:

This is a required question

Any other comments or questions on CONSORT EHEALTH


This is a required question

## STOP - Save this form as PDF before you click submit

To generate a record that you filled in this form, we recommend to generate a PDF of this page (on a Mac, simply select "print" and then select "print as PDF") before you submit it.
When you submit your (revised) paper to JMIR, please upload the PDF as supplementary file.
Don't worry if some text in the textboxes is cut off, as we still have the complete information in our database. Thank you!

## Final step: Click submit !

Click submit so we have your answers in our database!


|  |
| --- |
| Never submit passwords through Google Forms. |

Powered by


Google Forms

This content is neither created nor endorsed by Google.
  
Report Abuse
-
Terms of Service
-
Additional Terms

Screen reader support enabled.
